# Errors in visual search: How can we reduce them?

**DOI:** 10.3758/s13414-025-03095-6

**Published:** 2025-06-13

**Authors:** Aoqi Li, Jeremy M. Wolfe, Johan Hulleman

**Affiliations:** 1 The University of Manchester, Manchester, UK; 2 Brigham and Women’s Hospital, Boston, MA USA; 3 Harvard Medical School, Boston, MA USA

**Keywords:** Visual search, Error reduction, Stochastic errors, Deterministic errors

## Abstract

Observers routinely make errors in almost any visual search task. In previous online experiments, we found that indiscriminately highlighting all item positions in a noisy search display reduced errors. Here, we conducted two eye-tracking studies to investigate the mechanics of this error reduction: Does cueing direct attention to previously overlooked regions or enhance attention/processing at cued locations? Displays were presented twice. In Experiment 1, for half of the displays, the cue was only presented on the first copy (Cue – noCue) and for the other half, only presented on the second copy (noCue – Cue). Cueing successfully reduced errors but did not significantly affect reaction times (RTs). This contrasts with the online experiment where the cue increased RTs while reducing errors. In Experiment 2, we replicated the design of the online experiment by splitting the displays into noCue – noCue and noCue – Cue pairs. We now found that the cue reduced errors, but increased RTs on trials with high-contrast targets. The eye-tracking data show that participants fixated closer to items and fixation durations were shorter in cued displays. The smaller fixation-item distance reduced search errors, where observers never fixated the target, for low-contrast targets and the remaining low-contrast errors seemed to be recognition errors, where observers looked at the target but too quickly looked away. Taken together, these results suggest the main reason that errors were reduced was because attention was more properly directed to overlooked regions by the cues. Enhancement of attention at the cued areas may have played an auxiliary role.

**Supplementary Information:**

The online version contains supplementary material available at 10.3758/s13414-025-03095-6.

## Introduction

People constantly perform visual search tasks for a variety of purposes. For example, editors search for typos in manuscripts, custom officers search for contraband in luggage, security staff search for prohibited items in backpacks, radiologists search for abnormality in radiographs, etc. Even though people are usually clearly aware of what they should be looking for, it is almost inevitable that they will miss things from time to time. Such errors can have consequences, from confusing the reader (if a typo in a manuscript is missed) to injuring patient health (if an abnormality in a radiograph is missed). Therefore, it is of crucial importance to find ways of reducing such errors (Brady, 2017).

As one example, we can focus on misses in the field of radiology. Misses in radiology are especially interesting because of the threat to patients’ health and because those errors often have legal consequences (Berlin, 2007). The causes of radiological errors are often complex (Pinto & Brunese, 2010; Pinto et al., 2011), but it is worth looking into the underlying causes of errors in order to establish potential mitigation strategies (Onder et al., 2021). Broadly speaking, radiological errors have been categorized as perceptual errors (abnormality is not perceived) or cognitive errors (abnormality is perceived but misinterpreted), with perceptual errors accounting for 60–80% of radiologists’ errors (Berlin, 2014; Bruno et al., 2015; Caranci et al., 2015). Perceptual errors can be further subdivided into ‘search’ errors, where the observer never looks at the target location, and ‘recognition’ errors, where the observer looks at the target briefly, but moves on without apparently recognizing what they looked at (Kundel et al., 1978). We will make use of this distinction here. The common way for radiologists to reduce errors is somewhat atheoretic: Radiologists are encouraged to learn from errors, by recording their errors and attending error meetings (Mankad et al., 2009).

Traditionally, psychologists have also been interested in error reduction, including errors committed during the search process.

In this paper, we focus on miss errors in a simple T-vs.-L task with 50% prevalence. In our previous work, we used this task to assess the proportion of errors that were ‘stochastic’, i.e., errors occurring randomly with some probability from trial to trial as opposed to ‘deterministic’, i.e., errors that will be made every time a specific observer searches through a specific search display (Li et al., 2024). In those studies, we presented each search display twice in a randomly ordered set of trials. If errors are stochastic, then the probability of missing the target on both appearances of the search stimulus ($P12$) is given by the probability of missing the first appearance ($P1$) times the probability of missing the second ($P2$). If the errors are perfectly deterministic on the first and second appearances, then the probability of missing both is the same as the probability of missing the first appearance. We found that errors were essentially stochastic when search displays consisted of clearly visible Ts and Ls. When the task became more difficult, errors were a mix of stochastic and deterministic. With the difficult search displays that led to a mix of both types of errors, we also found that an intervention highlighting all item positions reduced miss errors and that this reduction mainly reduced deterministic errors. However, it should be noted that this reduction in miss errors came at the price of an increase in reaction times (RTs). Two other attempts at reducing the number of miss errors (a 500-ms yellow dot that highlighted random positions and a window that moved across the search display in a spiral motion) did not result in any improvements in search performance.

**Fig. 1:**
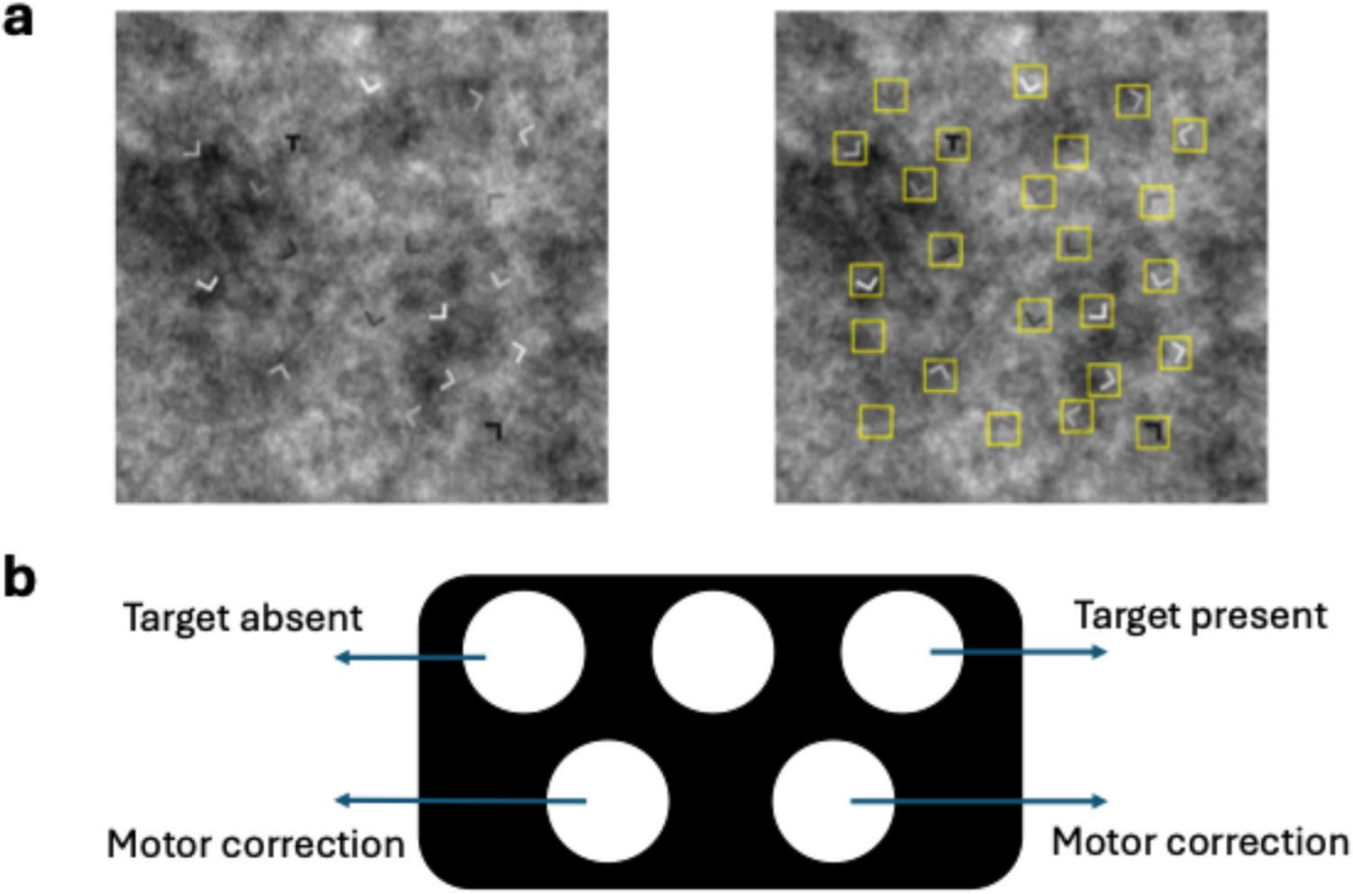
Stimuli and button box. (**a**) Stimuli. *Left:* A noCue stimulus. *Right:* A Cue stimulus. (**b**) Schematic of the button box used to register responses

Building on our previous work, in this paper we use eye tracking to investigate the mechanics of how cueing reduces errors. Consider a search for a T among Ls on a noisy background as shown in Fig. 1a. A cue surrounding all item locations (or all potential target locations) could reduce errors in either of two ways: (1) attention could be more effectively directed so that items that would have been overlooked in the uncued condition are more reliably attended in the presence of a cue, or (2) attention/processing could be effectively enhanced at the cued areas. More effective direction of attention would express itself as a reduction in search errors in cued trials: participants will look at locations that they skipped in uncued trials. Consequently, the remaining miss errors would predominantly be recognition errors. In eye-tracking terms this would imply a smaller distance between the nearest fixation and the target on miss trials. Enhanced processing should make participants better at recognizing the target. This implies that there will be fewer recognition errors, without a change in the distance between nearest fixation and target on miss trials. Please note that more effective direction and enhanced processing are not mutually exclusive: it could be the case that the cue allows both for fixation of previously skipped areas and for enhanced processing once attention has arrived. Our eye-tracking data demonstrate that the main effect of cueing appears to be better direction of attention, with fixations falling closer to items on cued trials and mostly recognition errors for low-contrast targets. However, shorter fixation durations in cued trials suggest that there may also be some enhanced processing. This is probably due to the fact that the yellow box surrounding the items in the cued trials reduced positional uncertainty. Moreover, better foveation of items in cued trials may also have led to enhanced processing. Nevertheless, the major driver for the reduction in miss rates in cued displays seems to have been the fixation of areas that were not fixated in uncued displays. Any enhanced processing was a byproduct of the better direction of attention.

## Experiment 1

### Method

#### Participants

For Experiment 1, we tested 20 participants (three males, 17 females, mean age = 19.6 years, SD = 0.8, min = 18, max = 21 years) from the BSc Psychology programme at the University of Manchester. We aimed for the same number of participants as in the online (Li et al., 2024) version of the experiment to maximize the comparability of the results. All participants reported normal or corrected-to-normal vision and gave their informed consent before they began the experiment. Participants received course credits for their participation. Ethics approval came from the University of Manchester (2023–18305–31346).

#### Stimuli and apparatus

Experiment 1 was programmed in Matlab with Psychtoolbox-3. The stimuli were displayed on a BenQ XL2420 T monitor with a refresh rate of 60 Hz. Participants sat with their heads immobilized in a chin rest at a distance of about 56 cm from the screen. One degree of visual angle corresponded to about 35 pixels on the screen. The display area was 360 mm $\times$ 270 mm (1280 $\times$ 960 pixels, 36.6 $^\circ$ $\times$ 27.4 $^\circ$). Eye movements were recorded using an EyeLink Portable Duo system (version: 6.5, SR Research Ltd, Ontario, Canada) with a sampling rate of 1,000 Hz. Gaze data were parsed into saccades and fixations by the EyeLink online parser. Velocity and acceleration thresholds were set as 35 (°/sec) and 8,000 (°/sec^2^). A nine-point calibration was completed before the first block and was validated before each block. When the validation failed, the eye tracker was calibrated again.

The stimuli consisted of 24 Ts and Ls against a background composed of $1/{f}^{1.3}$ noise, as shown in Fig. 1a. For each search array, the noisy background was randomly selected from ten noise images of 960 $\times$ 960 pixels (27.4 $^\circ$ $\times$ 27.4 $^\circ$). The length of vertical and horizontal lines of Ts and Ls was 30 pixels (0.86 $^\circ$). The orientations of the letters were randomly selected from [30, 60, 90, 120, 150, 180, 210, 240, 270, 300, 330, 360]. The minimum distance between any two letters was always larger than 0.12 image height (3.3 $^\circ$) to avoid overlapping. The target locations were evenly distributed across four spatial quadrants. The target contrast (defined by the difference between target greyscale and average background greyscale [T-B]) was controlled to be [−105, −75, −45, −15, 15, 45, 75, 105] using greyscale values from 0 (black) to 255 (white). The distractor greyscales were randomly drawn from [0, 255], so the theoretical range of distractor contrasts (letter ‘L’ to background ‘B’ or [L-B]) is [−255, 255], depending on the average background greyscale. In the following analyses, we used the absolute values of the differences as target contrasts or distractor contrasts for simplicity. All search arrays were generated before the experiment. Crossing target presence (2), T-B levels (8) and target locations (4) yielded 64 combinations (2*8*4). Six search arrays were generated for each parameter combination, resulting in a total of 384 stimuli. Since all stimuli were presented twice, the total number of trials was 768. All participants saw the same 768 search displays, but in different random orders.

#### Design and procedure

A button box (Fig. 1b) was used to register responses. Participants were instructed to press the upper right button if they found the target ‘T’ and the upper left if they did not. The stimulus presentation sequence was as follows: a fixation cross (1,000 ms) was followed by the search display. After their initial response, participants could press the bottom right or bottom left button within 1 s to reverse the response if they thought that they made a motor error. The search time was limited to 20 s. Please note that although a fixation cross was presented, triggering of the search display was not dependent on exact fixation location. Trial-by-trial feedback was not given, but the percentage correct was displayed at the end of each block. For half of the stimuli, the cue appeared on the second copy (noCue – Cue condition). For the other half of the stimuli, the cue appeared on the first copy (Cue – noCue condition). The cueing intervention was implemented by highlighting all the letters with yellow squares around them (size = 60 pixels, i.e., 1.7 $^\circ$) as shown in Fig. 1a (right). The experiment therefore had a design with three factors, each with two levels: repetition, condition and target. Participants were required to finish a 16-trial practice session before the experiment and would only be able to begin the experiment with an accuracy higher than 0.75, otherwise they had to repeat the practice. During practice, participants saw both cued and uncued displays,

#### Data exclusion

For the eye-tracking data, fixations (0.08%) and saccades (1.02%) that fell outside of the stimulus area were excluded. At trial level, trials with RTs smaller or greater than 2.5 SDs from the mean RT in each cell of the combination target $\times$ cue $\times$ repetition (1.93%) and trials where participants corrected their motor responses (1.87%) were removed for each observer. When one trial was removed, the other copy of the trial was also removed (92.70% remained). After the removal of the above trials, we further checked the d’ of all the participants. No participant was removed from Experiment 1 (min d’ = 2.78, max d’ = 4.99).

#### Manual responses

##### *Miss rates*

**Fig. 2:**
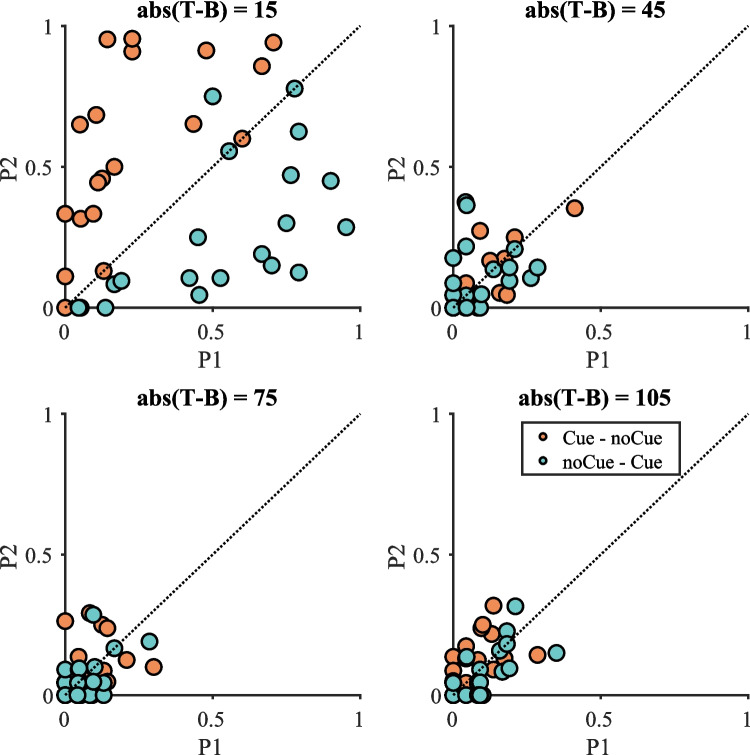
Miss rate data on target-present trials split by target contrast in Experiment 1. Orange datapoints above the line P1 = P2 and blue datapoints below indicate a positive effect of the cue

Figure 2 shows the impact of the cue on miss rate data when target-present trials were split by absolute target contrast (the results pooled over all target-present trials are presented in Fig. S1 in the Online Supplementary Material (OSM)). In each plot of Fig. 2, the second copy miss rate ($P2$) is plotted as a function of the first copy miss rate ($P1$). It seems that the cue was only effective when abs(T − B) = 15 as can be seen in the separation of the data points for the two conditions. For other values of abs(T − B), error rates are much lower and cluster around the P1 = P2 line. A three-way repeated-measures ANOVA with condition, repetition and target contrast ($2\times 2\times 4$) as within-subject factors was conducted on the miss rates. The effect of target contrast was significant [F(3, 57) = 39.10, p < 0.001, ${\eta }_{p}^{2}$ = 0.673], with higher miss rates on low-contrast targets. The interaction between repetition and condition was also significant [F(1, 19) = 31.41, p < 0.001, ${\eta }_{p}^{2}$ = 0.623], with smaller $P1$ in the Cue – noCue condition but smaller $P2$ in the noCue – Cue condition. This significant interaction between repetition and condition suggests that the cue effectively reduced errors. The three-way interaction between repetition, condition and target contrast was significant as well [F(3, 57) = 26.76, p < 0.001, ${\eta }_{p}^{2}$ = 0.585].

Due to the significant three-way interaction, four two-way repeated-measures ANOVAs with condition and repetition as within-subject factors were conducted for each target contrast separately. Results are presented in Table 1. The interaction between repetition and condition was significant for target contrast = 15. $P1$ was significantly smaller than $P2$ in the Cue – noCue condition [t(19) = 5.62, p < 0.001] while $P1$ was significantly larger than $P2$ in the noCue – Cue condition [t(19) = 4.74, p < 0.001]. The effects for the higher contrasts were either non-significant or failed to break the Bonferroni-corrected critical value of 0.0125.

**Table 1:** Results of the repeated-measures ANOVA on miss rates for each target contrast in Experiment 1 (Bonferroni-corrected α = 0.0125)

Target contrast	Effect	$F$	$p$	${\eta }_{p}^{2}$
15	Repetition	F(1, 19) = 1.12	0.303	0.056
	Condition	F(1, 19) = 1.05	0.319	0.052
	Repetition * condition	F(1, 19) = 35.07	< 0.001	0.649
45	Repetition	F(1, 19) = 0.0575	0.813	0.003
	Condition	F(1, 19) = 0.1578	0.696	0.008
	Repetition * condition	F(1, 19) = 2.0805	0.165	0.099
75	Repetition	F(1, 19) = 0.0112	0.917	0.001
	Condition	F(1, 19) = 1.2081	0.285	0.060
	Repetition * condition	F(1, 19) = 0.4529	0.509	0.023
105	Repetition	F(1, 19) = 0.682	0.419	0.035
	Condition	F(1, 19) = 0.373	0.549	0.019
	Repetition * condition	F(1, 19) = 6.241	0.022	0.247

The miss rate results clearly show that cueing only effectively reduced errors on low-contrast targets. This replicates the results of our previous online experiments that the intervention highlighting all item positions succeeded in reducing errors. There are two potential reasons for this success: (1) The cueing could have directed observers to low contrast-targets that would otherwise have been overlooked, or (2) the cueing could have improved processing at locations that were attended even without a cue but were inadequately processed.

##### *Reaction times (RTs)*

**Fig. 3:**
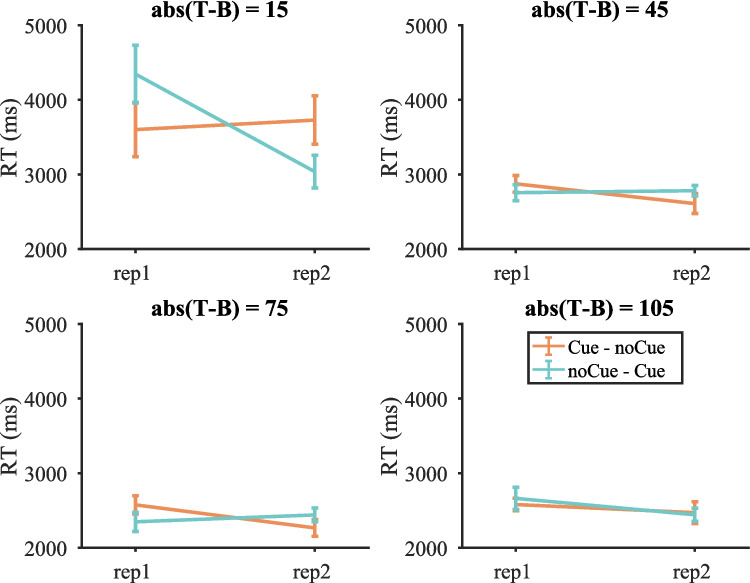
Mean reaction times (RTs) on target-present trials split by target contrast in Experiment 1. Error bars represent $\pm 1$ standard error

For the analysis of RT data, we only included the cases where participants responded correctly to both occurrences of a stimulus (these trials accounted for 86.75% of the trials used in the miss-rate analysis). Figure 3 shows RTs on target-present trials split by target contrast (RTs pooled over all target-present trials and RTs on target-absent trials are shown in Fig. S2 (OSM)). Similar to the miss rates, the cue seemed to mainly affect RTs on low-contrast targets with abs(T − B) = 15. We then performed a three-way repeated-measures ANOVA with condition, repetition and target contrast as within-subject factors. Three participants were excluded due to empty cells (e.g., some participants failed to respond correctly twice on any low-contrast target). The two-way interaction between condition and repetition was not significant [F(1, 16) = 0.65, p = 0.430, ${\eta }_{p}^{2}$ = 0.039], but the three-way interaction between condition, repetition and target contrast was [F(3, 48) = 8.64, p < 0.001, ${\eta }_{p}^{2}$ = 0.351]. Therefore, four two-way repeated-measure ANOVAs with cue and repetition as within-subject factors were conducted for each target contrast separately. Table 2 presents the results. All effects were either non-significant or failed to break the Bonferroni-corrected critical value of 0.0125.

**Table 2:** Results of the repeated-measures ANOVA on reaction times (RTs) for each target contrast in Experiment 1 (Bonferroni-corrected α = 0.0125)

Target contrast	Effect	$F$	$p$	${\eta }_{p}^{2}$
15	Repetition	F(1, 16) = 5.1153	0.038	0.242
	Condition	F(1, 16) = 0.0129	0.911	0.001
	Repetition * condition	F(1, 16) = 6.0540	0.026	0.275
45	Repetition	F(1, 16) = 2.5833	0.128	0.139
	Condition	F(1, 16) = 0.0988	0.757	0.006
	Repetition * condition	F(1, 16) = 1.4553	0.245	0.083
75	Repetition	F(1, 16) = 1.820	0.196	0.102
	Condition	F(1, 16) = 0.106	0.749	0.007
	Repetition * condition	F(1, 16) = 2.404	0.141	0.131
105	Repetition	F(1, 16) = 1.7485	0.205	0.099
	Condition	F(1, 16) = 0.0917	0.766	0.006
	Repetition * condition	F(1, 16) = 0.2054	0.657	0.013

This means that the cue did not significantly affect RTs, although RTs tended to become shorter on Cue trials for target contrast = 15 whereas the effect of cue was in the opposite direction for higher contrasts. This was different from the RT pattern in our previous online Experiment 3c, where the cue slowed the search (Li et al., 2024). For comparison, we also split the trials from the previous online experiment by target contrast. As shown in Fig. S3 (OSM), the cue seemed to increase RTs for all contrasts. Statistics in Tables S1–S4 (OSM) suggest that the effect of the cue was only significant for target contrast = 15 (though only six participants with data in each cell remained for target contrast = 15).

#### Eye movements

**Fig. 4:**
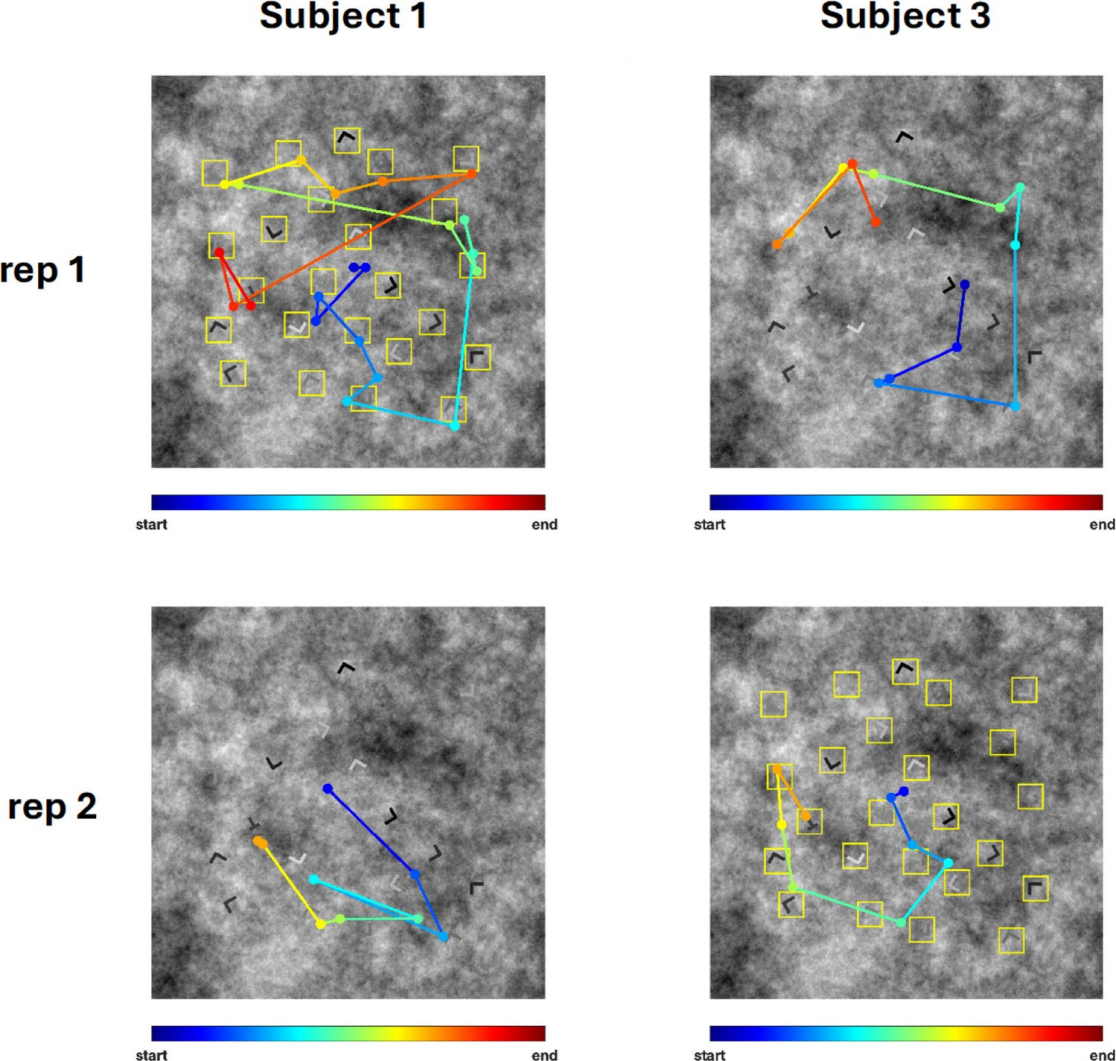
Scanpath visualization for one of the stimuli in Experiment 1. Each dot represents a fixation. The colours of the dots indicate the fixation order. For Subject 1, the stimulus was in the Cue – noCue condition. For Subject 3, it was in the noCue – Cue condition

Figure 4 visualizes the scanpaths for two observers on one of the stimuli in Experiment 1. For Subject 1, the Cue stimulus appeared first, followed later in the sequence by the noCue stimulus. The situation was reversed for Subject 3. In these examples, the number of fixations seems to be more affected by repetition rather than by the presence of the cue but, of course, these are just illustrative examples. To get a more systematic picture of how the cue influenced eye movements, fixation-item distance and three basic eye-movement measures were analysed: number of fixations, fixation durations and saccade length. In the following analyses on these three basic measures (which included all fixations and saccades made during a trial), we only included the stimuli to which participants responded correctly twice as we did for the RT analyses. The analyses of the basic eye-movement measures are grouped by target contrast since distractor contrasts were randomly chosen and can therefore not be used as grouping factor.

##### *Fixation-item distance*

 As noted above, based on the classic work of Kundel et al. (1978) in radiology, miss errors have been classified as ‘search’ errors or as ‘recognition’ errors based on whether the target was fixated or not during a trial. A target that is never fixated is considered to be a search error. A target that is briefly fixated but not reported is a recognition error. A target that is fixated for an extended period of time would be labelled as a ‘decision error’ but those are vanishingly rare in this task. Unfortunately, application of this scheme is problematic in our experiment because of the varying item contrasts. A fixation that is 2 degrees away from the centre of a target might be adequate to identify a high-contrast T but not a low-contrast T. We therefore adopted a somewhat different method of analysis to examine the effect of the cues on visual search. In our analysis, we look at the following metrics.

- **N fixation**: Where are Observers (Os) fixating when they find the target? Taking the last five fixations before the end of a hit trial, we take the fixation closest to the target as the target fixation as long as the preceding saccade is greater than 1 degree in length. If it is less than 1 degree, we take that short saccade as a corrective saccade and take its origin as the target fixation. We look at the last five fixations because Os sometimes fixate the target and then move their eyes elsewhere while making a response.^1^
- **N-1 fixation:** Where were Os fixating on the fixation prior to the target fixation? For uncued displays we assume that they detected the target from that location and then moved to the target. For cued displays this assumption probably does not hold, since Os may simply be making a saccade towards a yellow cue box, unaware that it contains a target.
- **Miss trial minimum distance:** How close did Os get to the target location on trials where they failed to detect the target?

**Fig. 5:**
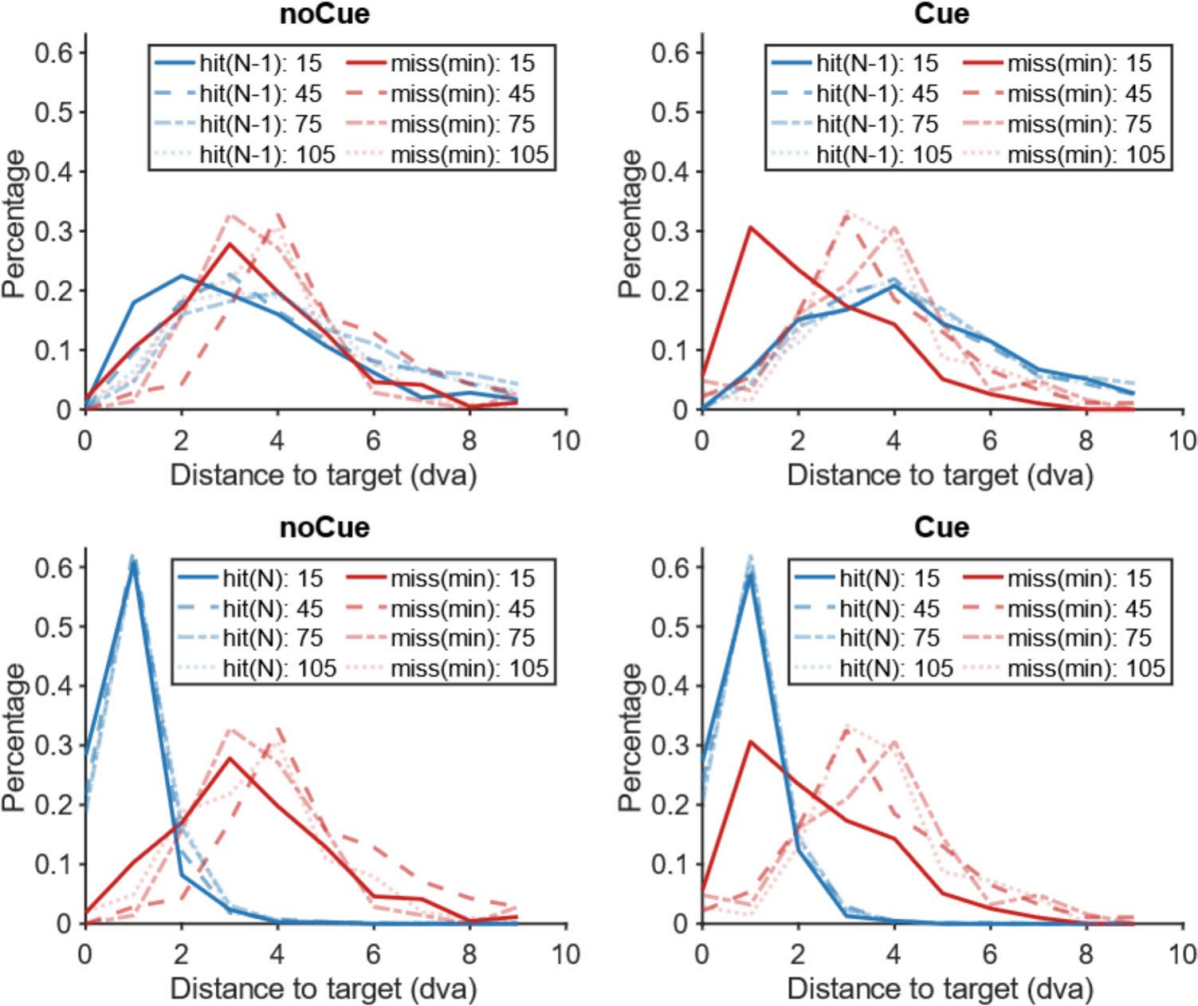
Distributions of the distance between target and fixation in Experiment 1 as a function of target contrast (15, 45, 75, 105), cue (cue, nocue), fixation (N-1, N), and accuracy (hit, miss). **Top:** N-1 fixations. **Bottom:** N-fixations. **Left**: no-cue trials. **Right**: cue trials. Blue: hit trials; Red: miss trials (please note that this is the closest fixation to the target across the trial. Hence, there is no distinction between N and N-1 fixations). Solid lines: target contrast 15; large dashed lines: target contrast 45; small dashed lines: target contrast 75; dotted lines: target contrast 105

Our specific interest is in how these measures changed with and without the cue. If the cue enables more effective direction of attention, we expect that the miss trial minimum distance would be reduced since most errors will be recognition errors, rather than search errors. We can also expect the N-1 fixations to be further from low-contrast targets, since Os are no longer dependent on actual detection of the target. Figure 5 shows the normalized histograms of these measures for various contrasts. From Fig. 5 it becomes clear that it is the lowest contrast targets (contrast 15) where the effects of the cue are seen most prominently.

The top row of Fig. 5 shows that, on hit trials (blue lines), the cue produced saccades to low-contrast targets that came from further away because the cue, itself, could attract attention. As a consequence, the distribution of N-1 fixations on cue trials is shifted to longer distances for the low-contrast targets (solid blue line). For the higher contrast targets the difference between cued and uncued trials is much smaller (other blue lines). On miss trials (red lines) on the other hand the participants fixated closer to low-contrast targets on cue trials. The peak in the distribution of minimum distance of fixation to target is shifted to the left for the low-contrast targets (solid red line). Again, this effect is much less prominent for the higher contrast targets (other red lines).

The bottom row of Fig. 5 shows that the cue did not really have an effect on the N fixations: irrespective of the presence of a cue, participants liked to closely fixate the target when they gave a present response (blue lines). What is noticeable though is that in the misses for noCue trials (red lines), the effect of target contrast was fairly minimal: the distributions of the distance between the closest fixation and the target largely overlap. Moreover, the peaks of these distributions are further away from the target than the actual N-fixations (blue lines). This suggests that the participants never laid eyes on the target during miss trials without a cue. For the cue trials, a shift towards the target happened for low-contrast targets (solid red line). Here, the distribution of the minimum distance between fixation and target on miss trials is now much more similar to that of hit trials (blue lines). For higher contrast targets that were missed (other red lines), the addition of a cue affected the distribution of distance between closest fixation and target much less. So, it seems that the addition of the cue turned the misses of low-contrast targets into recognition errors, whereas the misses of higher contrast targets remained search errors.

It is not unusual for the eyes to fall near a target without the target being recognized. Wu and Wolfe (2022) reported that, even when the eyes were very close to the target, the next fixation went to the target on only about 50% of instances. Most of these trials became hit trials in the end because the eyes returned to the vicinity of the target at some later point and the target was successfully detected. The same pattern is seen in our data, as can be seen in Fig. S4 (OSM).

**Fig. 6:**
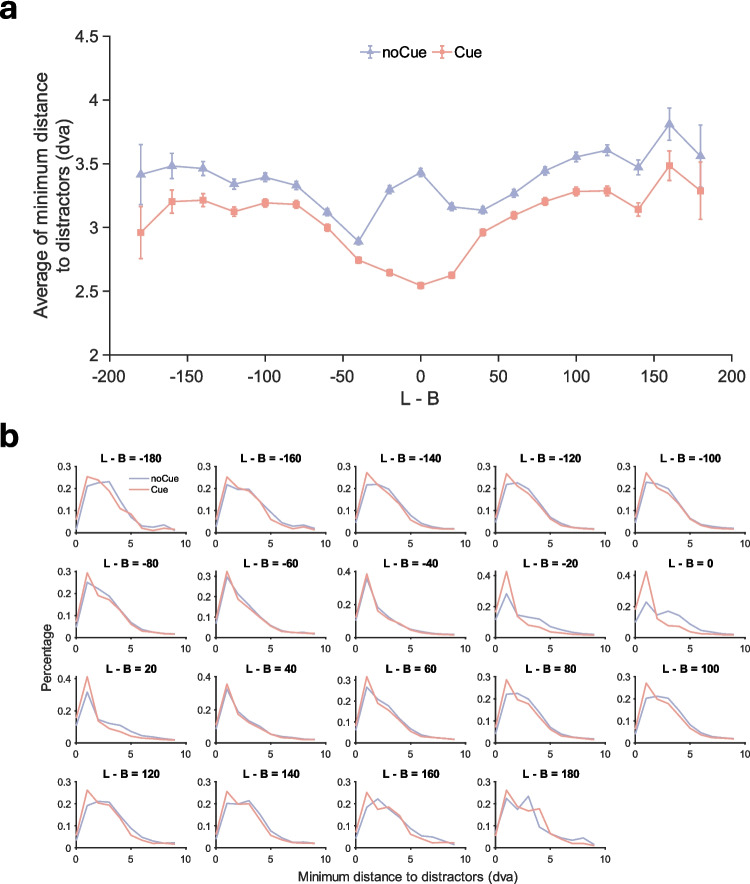
Distance between fixations and distractors in Experiment 1. (**a**) Binned average of minimum fixation-distractor distance as a function of distractor contrast in Experiment 1. Bin width = 20. Error bars represent $\pm 1$ standard error. (**b**) Distributions of minimum fixation-distractor distance in Experiment 1 for each contrast bin

In addition to the analysis of fixation-target distance, we further analysed the binned average of minimum distance between any distractor and any fixation as a function of distractor contrast.^2^ Fig. 6a shows the results. When there was no cue, the minimum fixation-item distance first decreased with item contrast [abs(L—B)] but increased again at extremely low contrast, indicating that observers did not notice the existence of some low-contrast items in the absence of the cue. However, when the cue was present, this bump in the data disappears. Now, the distance monotonically decreases with item contrast, suggesting that low-contrast items simply demanded more precise fixation. In addition, the minimum fixation-item distance was lower in the presence of the cue, especially for those low-contrast items.

Figure 6b shows the distributions of minimum fixation-distractor distance as a function of item contrast (L-B), which also suggests the cue effect was stronger for low-contrast distractors. At the higher contrasts, the cue and no-cue distributions are similar. At low contrast, (L − B = – 20, 0 and 20), it can be seen that the presence of the cue shifts the minimum distances to shorter values. Therefore, the cue seems to have guided the eyes and attention to areas that might have been completely overlooked, providing an explanation of how the cue reduced errors.

##### *Number of fixations*

**Fig. 7:**
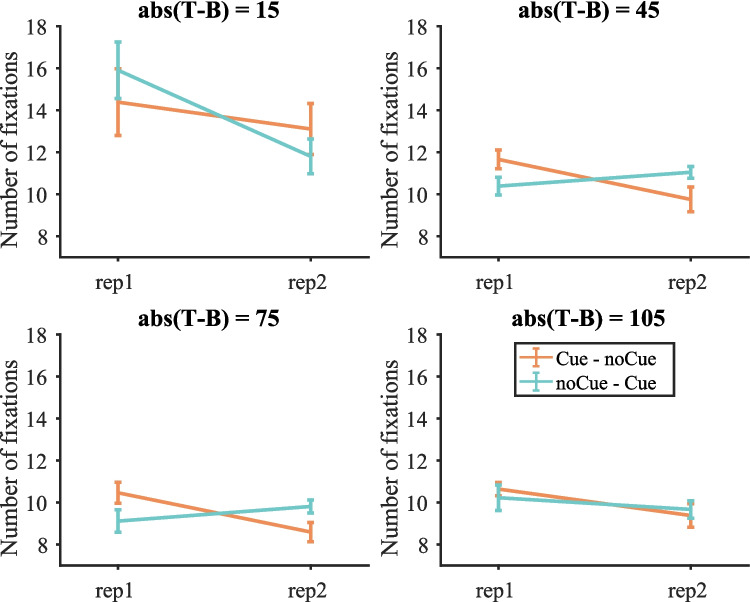
Number of fixations on target-present trials split by target contrast in Experiment 1. Error bars represent $\pm 1$ standard error

Figure 7 shows the number of fixations on target-present trials split by target contrast (the number of fixations on target-present and target-absent trials is shown in Fig. S5 (OSM)). We then conducted a three-way repeated-measures ANOVA with condition, repetition and target contrast as within-subject factors on the number of fixations The same three participants as before were excluded due to empty cells. The two-way interaction between condition and repetition was not significant [F(1, 16) = 0.62, p = 0.444, ${\eta }_{p}^{2}$ = 0.037], but the three-way interaction between condition, repetition and target contrast was [F(3, 48) = 5.74, p = 0.002, ${\eta }_{p}^{2}$ = 0.264]. Therefore, four two-way repeated-measure ANOVAs with condition and repetition as within-subject factors were conducted for each target contrast separately. As shown in Table 3, all effects were either non-significant or failed to break the Bonferroni-corrected critical value of 0.0125.

**Table 3:** Results of the repeated-measures ANOVA on fixation number for each target contrast in Experiment 1 (Bonferroni-corrected α = 0.0125)

Target contrast	Effect	$F$	$p$	${\eta }_{p}^{2}$
15	Repetition	F(1, 16) = 6.7366	0.020	0.296
	Condition	F(1, 16) = 0.0156	0.902	0.001
	Repetition * condition	F(1, 16) = 1.7632	0.203	0.099
45	Repetition	F(1, 16) = 5.9793	0.026	0.272
	Condition	F(1, 16) = 0.0011	0.974	0.000
	Repetition * condition	F(1, 16) = 7.1707	0.017	0.309
75	Repetition	F(1, 16) = 3.7511	0.071	0.190
	Condition	F(1, 16) = 0.0403	0.843	0.003
	Repetition * condition	F(1, 16) = 6.8440	0.019	0.300
105	Repetition	F(1, 16) = 3.9790	0.063	0.199
	Condition	F(1, 16) = 0.0328	0.859	0.002
	Repetition * condition	F(1, 16) = 0.7637	0.395	0.046

Similar to the results of RTs, the cue did not significantly affect the number of fixations. But numerically, the effect of the cue was different for target contrast 15 (pushing the number of fixations down) and the other target contrasts (pushing the number of fixations up), suggesting that participants searched more efficiently with fewer fixations for low-contrast targets.

##### *Fixation durations*

**Fig. 8:**
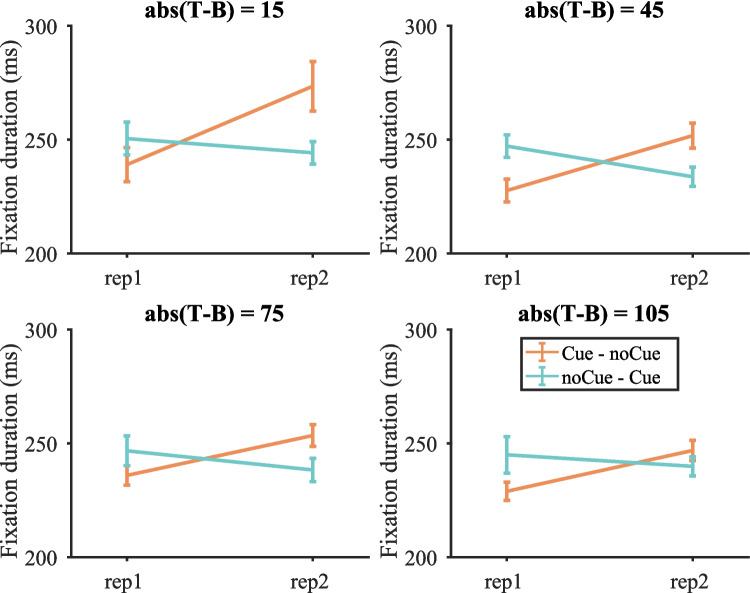
Fixation durations on target-present trials split by target contrast in Experiment 1. Error bars represent $\pm 1$ standard error

Figure 8 shows the fixation durations on target-present trials split by target contrast (fixation durations on target present and target-absent trials are shown in Fig. S6 (OSM)). A three-way repeated-measures ANOVA with condition, repetition and target contrast as within-subject factors was conducted. As before, three participants were excluded due to empty cells. The effects of repetition and target contrast were significant [repetition: F(1, 16) = 20.43, p < 0.001, ${\eta }_{p}^{2}$ = 0.561; target contrast: F(3, 48) = 3.33, p = 0.027, ${\eta }_{p}^{2}$ = 0.172], but the effect of condition was not [F(1, 16) = 0.49, p = 0.496, ${\eta }_{p}^{2}$ = 0.030]. The interaction between condition and repetition was significant [F(1, 16) = 61.40, p < 0.001, ${\eta }_{p}^{2}$ = 0.793]. For the Cue – noCue condition, fixation durations were shorter on the first copy of stimuli. For the noCue – Cue condition, fixation durations were shorter on the second copy. Other two-way interactions and the three-way interaction were not significant [repetition $\times$ target contrast: F(3, 48) = 2.14, p = 0.107, ${\eta }_{p}^{2}$ = 0.118; condition $\times$ target contrast: F(3, 48 = 1.94, p = 0.136, ${\eta }_{p}^{2}$ = 0.108; repetition $\times$ condition $\times$ target contrast: F(3, 48) = 1.47, p = 0.234, ${\eta }_{p}^{2}$ = 0.084]. This suggests that participants made shorter fixations on Cue trials regardless of target contrast.

##### *Saccade length*

**Fig. 9:**
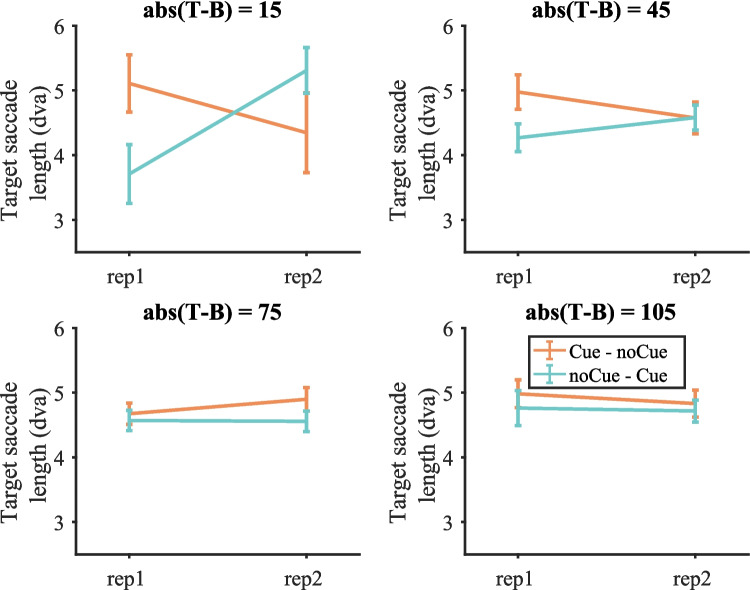
Target saccade length on target-present trials split by target contrast in Experiment 1. Error bars represent $\pm 1$ standard error

Both search saccades and target saccades were analysed. As previously, a target saccade was defined as the saccade before a target fixation. Search saccades include all the saccades on target-absent trials and all the saccades before the target saccade on target-present trials. Figure 9 shows the average length of target saccades on target-present trials split by target contrast (target saccade length pooled over all target-present trials is shown in Fig. S7 (OSM)). A three-way repeated-measures ANOVA with condition, repetition and target contrast as within-subject factors was conducted on target saccade length. Again, three participants were excluded due to empty cells. For target saccade length, all main effects were not significant. The interaction between condition and repetition was significant [F(1, 16) = 10.41, p = 0.005, ${\eta }_{p}^{2}$ = 0.394]. The other two-way interactions were not significant. The three-way interaction between condition, repetition and target contrast was significant [F(3, 48) = 4.47, p = 0.008, ${\eta }_{p}^{2}$ = 0.219].

Based on the significant three-way interaction, two-way repeated-measures ANOVAs on target saccade length with condition and repetition as within-subject factors were conducted for each target contrast separately. Results are presented in Table 4. All effects and interactions were either non-significant or failed to break the Bonferroni-corrected critical value of 0.0125, though there was a trend (p = 0.017) for longer target saccades on Cue trials when target contrast = 15.

**Table 4:** Results of the repeated-measures ANOVA on target saccade length for each target contrast in Experiment 1 (Bonferroni-corrected α = 0.0125)

Target contrast	Effect	$F$	$p$	${\eta }_{p}^{2}$
15	Repetition	F(1, 16) = 0.770	0.393	0.046
	Condition	F(1, 16) = 0.225	0.641	0.014
	Repetition * condition	F(1, 16) = 7.148	0.017	0.309
45	Repetition	F(1, 16) = 0.0337	0.857	0.002
	Condition	F(1, 16) = 4.0002	0.063	0.200
	Repetition * condition	F(1, 16) = 3.2235	0.091	0.168
75	Repetition	F(1, 16) = 0.527	0.478	0.032
	Condition	F(1, 16) = 5.040	0.039	0.240
	Repetition * condition	F(1, 16) = 0.598	0.451	0.036
105	Repetition	F(1, 16) = 0.404	0.534	0.025
	Condition	F(1, 16) = 0.728	0.406	0.044
	Repetition * condition	F(1, 16) = 0.164	0.691	0.010

Figure S8 (OSM) shows search saccade length on target-present and target-absent trials. Figure S9 (OSM) shows search saccade length on target-present trials split by target contrast. Statistics suggest that the cue did not significantly affect search saccade length. Taken together, the cue mainly increased target saccade length for low-contrast targets (target contrast = 15), though the effects were not significant. This suggests that with the help of the cue observers could recognize targets at a larger distance from fixations. This might partly explain why the cue could effectively reduce miss errors on low-contrast targets.

## Experiment 1: Discussion

In Experiment 1, the stimuli were split into a Cue – noCue condition and a noCue – Cue condition. We found that the cue reduced miss errors on low-contrast targets but did not significantly affect RTs. Eye-tracking data suggest that the cue reduced the minimum distance between any item and its closest fixation, especially for low-contrast items. This reduced low-contrast search errors and the remaining low-contrast errors seemed to be predominantly recognition errors. For other basic eye-tracking measures, the cue did not significantly affect the number of fixations. Fixation durations were significantly reduced by the cue regardless of target contrast. Effects of cue on target saccade length and search saccade length were not significant, though target saccade length was numerically increased by the cue on trials with low-contrast targets. Whether the cue was present or not, the errors were a mix of stochastic and deterministic errors (see Fig. 17 in the Appendix).

The one thing that stands out is that, in Experiment 1, the cue reduced miss errors on low-contrast targets but did not significantly affect RTs. Numerically, RTs were even faster for low-contrast targets in cue displays. This deviates from our previous online Experiment 3c (Li et al., 2024), where the cue also reduced miss errors, but increased RTs. The discrepancy might be due to differences in experiment design. For instance, in Experiment 1 half of the trials were cued, whereas in the online experiment only 25% of the trials were cued. Moreover, in Experiment 1, the cued trials were equally likely throughout the experiment, whereas in the online experiments they became more likely towards the end of the experiment. This may have affected the way that the online participants interacted with the cues. Therefore, in Experiment 2, we attempted to mirror the previous online Experiment 3c more closely by splitting the stimuli into a noCue – noCue condition and a noCue – Cue condition.

## Experiment 2

### Method

#### Participants

For Experiment 2, we tested 20 participants (three males, 17 females, mean age = 19.6 years, SD = 1.1, min = 18, max = 22 years) from the BSc Psychology programme at the University of Manchester. All participants reported normal or corrected-to-normal vision and gave their informed consent before they began the experiment. Participants received course credits for their participation. Ethics approval came from the University of Manchester (2023–18305–31346).

#### Stimuli and apparatus

The stimuli and apparatus were the same as in Experiment 1 except that we eliminated target-present stimuli with T − B = $\pm$ 105 to reduce the burden on the participants, since Experiment 2 contained 75% no-Cue trials across its two conditions. A corresponding percentage of target-absent stimuli were also removed. Thus, the total number of unique stimuli in Experiment 2 was 288, resulting in 576 trials since each was shown twice.

#### Design and procedure

The design and procedure were the same as in Experiment 1 except that the stimuli were now split into a noCue – noCue condition and a noCue – Cue condition. In the noCue – noCue condition, neither of the copies had the yellow box cues. In the noCue – Cue condition, there were no cues on the first copy but there were cues on the second copy. Participants were required to finish a 12-trial practice session before the experiment and were only able to begin the experiment with an accuracy higher than 0.75, otherwise, they had to repeat the practice.

#### Data exclusion

For eye-tracking data, fixations (0.24%) and saccades (1.15%) that fell outside of the stimulus area were excluded. At trial level, trials with RTs smaller or greater than 2.5 SDs from the mean RT in each cell of the combination target $\times$ cue $\times$ repetition (2.20%) and trials where participants corrected their motor responses (1.35%) were removed for each observer. When one trial was removed, the other copy of the trial was also be removed. After the removal of these trials, 93.21% remained. We further checked the d’ of all the participants. Two participants with d’ lower than 1.0 were removed (for the remaining participants, min d’ = 2.50, max d’ = 4.66).

#### Manual responses

##### *Miss rates*

**Fig. 10:**
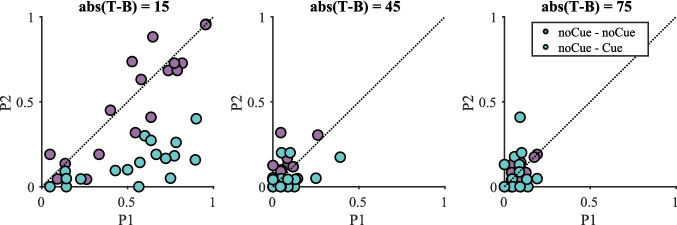
Miss rates on target-present trials split by target contrast in Experiment 2

Figure 10 shows miss rate data on target-present trials split by target contrast (the results pooled over all target-present trials are presented in Fig. S10 (OSM)). Please note that there were no differences between the miss rates for NoCue-NoCue and NoCue-Cue on first presentation (the purple and green dots are equally distributed along the x-axis of Fig. 10). Again, it seems that the cue was only effective when abs(T–B) = 15. We conducted a three-way repeated-measures ANOVA on the miss rate data for target-present trials with condition, repetition and target contrast as within-subject factors. The three-way interaction among condition, repetition and target contrast was significant [F(2, 34) = 24.1, p < 0.001, ${\eta }_{p}^{2}$ = 0.586], so three two-way repeated-measure ANOVAs with condition and repetition as within-subject factors were conducted for each target contrast separately (see Table 5). When target contrast = 15, the effects of condition and repetition were significant, as was the interaction between the two factors. Follow-up *t*-tests suggest that the miss rates were significantly lower on the second copy than the first copy in the noCue – Cue condition [*t*(17) = 7.63, p < 0.001] while there was no significant difference between the two copies in the noCue – noCue condition. When target contrast = 45 or 75, none of the effects or interactions were significant. There was only an effect of cue for low-contrast targets (target contrast = 15). This replicates the finding in Experiment 1.

**Table 5:** Results of the repeated-measures ANOVA on miss rates for each target contrast in Experiment 2 (Bonferroni-corrected α = 0.0167)

Target contrast	Effect	$F$	$p$	${\eta }_{p}^{2}$
15	Repetition	F(1, 17) = 50.0	< 0.001	0.746
	Condition	F(1, 17) = 48.7	< 0.001	0.741
	Repetition * condition	F(1, 17) = 34.4	< 0.001	0.669
45	Repetition	F(1, 17) = 0.355	0.559	0.020
	Condition	F(1, 17) = 0.523	0.479	0.030
	Repetition * condition	F(1, 17) = 2.474	0.134	0.127
75	Repetition	F(1, 17) = 0.0681	0.797	0.004
	Condition	F(1, 17) = 0.0508	0.824	0.003
	Repetition * condition	F(1, 17) = 0.1560	0.698	0.009

##### ***RTs***

**Fig. 11:**
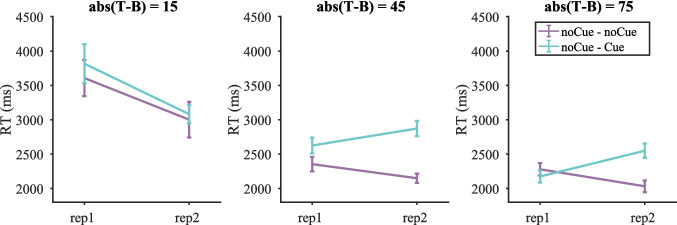
Reaction times (RTs) on target-present trials split by target contrast in Experiment 2. Error bars represent $\pm 1$ standard error

For the RT data analysis, we only included the stimuli to which participants responded correctly twice (these trials accounted for 85.71% of the remaining trials used in the miss-rate analysis). Figure 11 shows RTs on target-present trials split by target contrast (RTs pooled over all target-present trials and RTs on target-absent trials are shown in Fig. S11 (OSM)). A three-way repeated-measures ANOVA with condition, repetition and target contrast as within-subject factors was conducted on the RT data. One participant was excluded due to empty cells (i.e., the failure to respond correctly twice on any trial in some cells). The three-way interaction was significant [F(2, 32) = 3.53, p = 0.041, ${\eta }_{p}^{2}$ = 0.181], so three two-way repeated-measures ANOVAs with condition and repetition as within-subject factors were conducted for each target contrast separately.

Table 6 presents the results of the two-way ANOVA for each target contrast. For target contrast = 15, participants responded significantly faster on the second copy of trials, but the effect of condition and the interaction between condition and repetition were not significant, suggesting that the decrease in RTs from round 1 to round 2 were merely due to the effect of time in trial (repetition or practice) instead of the cue. For target contrast = 45, the effect of repetition was not significant, but the effect of condition was. Participants responded faster on the noCue – noCue condition than the noCue – Cue condition. The interaction between repetition and condition was also significant, with a larger effect of condition in round 2 (noCue versus Cue) than in round 1 (noCue versus noCue). This suggests that the increase in RT difference between noCue – noCue trials and noCue – Cue trials in round 2 was due to the presence of the cue. For target contrast = 75, the effect of repetition was not significant. The effect of condition was: RTs were faster for the noCue – noCue condition than the noCue – Cue condition. The interaction between repetition and condition was also significant, indicating that the RT difference between the two conditions was caused by the presence of the cue in round 2, with slower round 2 RTs for noCue – Cue than for noCue – noCue.

**Table 6:** Results of the repeated-measures ANOVA on reaction times (RTs) for each target contrast in Experiment 2 (Bonferroni-corrected α = 0.0167)

Target contrast	Effect	$F$	$p$	${\eta }_{p}^{2}$
15	Repetition	F(1, 16) = 14.811	0.001	0.481
	Condition	F(1, 16) = 1.145	0.300	0.067
	Repetition * condition	F(1, 16) = 0.234	0.635	0.014
45	Repetition	F(1, 16) = 0.0736	0.790	0.005
	Condition	F(1, 16) = 60.7979	< 0.001	0.792
	Repetition * condition	F(1, 16) = 8.9744	0.009	0.359
75	Repetition	F(1, 16) = 0.639	0.436	0.038
	Condition	F(1, 16) = 10.822	0.005	0.403
	Repetition * condition	F(1, 16) = 14.127	0.002	0.469

The cue did not significantly affect RTs on low-contrast targets (target contrast = 15), but increased RTs when target contrast = 45 or 75. Therefore, Experiment 2 partly replicated the RT pattern in our previous online experiment 3c (Li et al., 2024) where the cue slowed down the search. However, the decrease in miss errors mainly occurred on low-contrast targets, so the increase in RTs on high-contrast targets still does not explain why the cue reduced miss rates for low-contrast targets without a detriment to the RTs.

#### Eye movements

Fixation-distance as well as three main types of eye-movement measures (number of fixations, fixation durations and saccade length) were analysed. In the following analyses of the three basic measures, we only included stimuli to which participants responded correctly twice.

##### *Fixation-item distance*

**Fig. 12:**
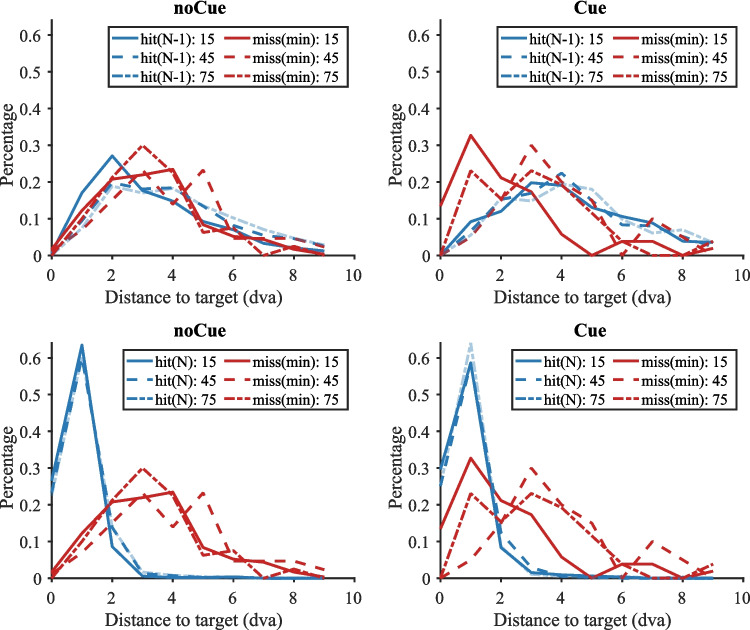
Distributions of the distance between target and fixation in Experiment 2 as a function of target contrast (15, 45, 75), cue (cue, nocue), fixation (N-1, N), and accuracy (hit, miss). **Top:** N-1 fixations. **Bottom:** N-fixations. **Left:** no cue trials. **Right:** cue trials. Blue: hit trials; Red: miss trials (please note that this is the closest fixation to the target across the trial. Hence, there is no distinction between N and N-1 fixations). Solid lines: target contrast 15; large dashed lines: target contrast 45; small dashed lines: target contrast 75

Figure S12 (OSM) shows the probability of the next fixation being the target fixation as a function of fixation-target distance in Experiment 2, which was similar to the results in Experiment 1. Then we compared the distance from fixation to target between hit trials and miss trials as shown in Fig. 12. The pattern is basically the same as in Experiment 1 (Fig. 5): The top row shows that the presence of a cue yielded a shift in the distance between N-1 fixation and target, especially for low-contrast targets (solid blue line). On miss trials, the nearest fixation fell closer to the target both for the low contrast (solid red line) and the highest contrast (small dashed red line). The bottom row shows that the cue had no influence on the N fixations (solid blue lines) which fell close to the target irrespective of cue and contrast. Again, the effect of target contrast was very limited on the misses for noCue trials (red lines). As before, the peaks of the miss distributions (red lines) are further away from the target than the peaks of the N-fixations (blue lines), indicating that the participants did not come close to the target during misses. For the cued trials, there was a shift towards the target both for low-contrast misses (solid red line) and for higher contrast misses (small dashes red line). This suggests that for these targets (contrast = 15 and contrast = 75), the cue turned search errors into recognition errors.

**Fig. 13:**
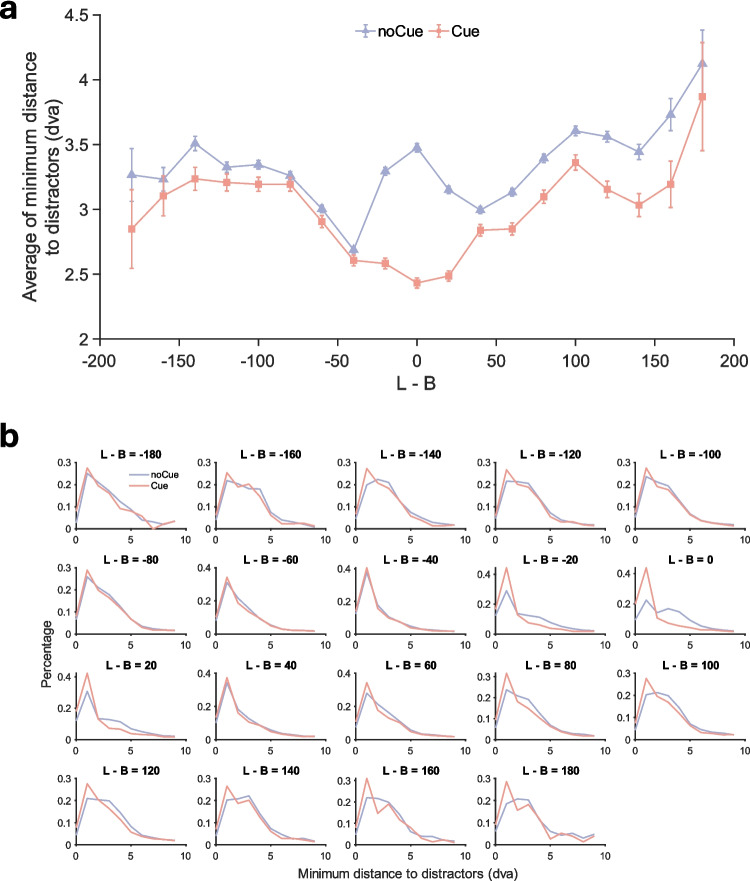
Distance between fixations and distractors in Experiment 2. (**a**) Binned average of minimum fixation-distractor distance as a function of distractor contrast in Experiment 2. Bin width = 20. Error bars represent $\pm 1$ standard error. (**b**) Distributions of minimum fixation-distractor distance in Experiment 2 for each contrast bin

Figure 13a shows the distance between any distractor and the nearest fixation as a function of distractor contrast, and basically replicates the patterns from Experiment 1. Without the cueing intervention, the distance between distractor item and closest fixation decreased from high contrast to low contrast but increased again at extremely low contrast. With the cueing intervention, the distance monotonically decreased with item contrast. The cue consistently decreased the distance, especially for those low-contrast items. Figure 13b shows the distributions of minimum fixation-distractor distance. As in Experiment 1, the cue effect was stronger for low-contrast distractors (L − B = – 20, 0 and 20). Therefore, the cue seems to have guided attention to low-contrast items that might have been completely overlooked without it, thereby reducing the number of missed low-contrast targets.

##### *Number of fixations*

**Fig. 14:**
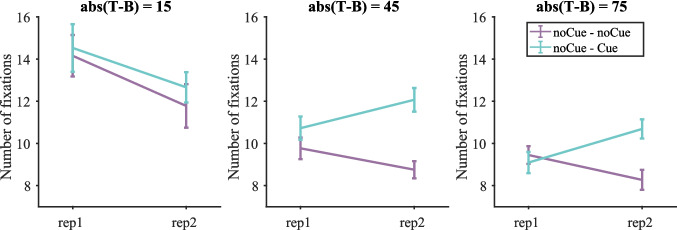
Number of fixations on target-present trials split by target contrast in Experiment 2. Error bars represent $\pm 1$ standard error

Figure 14 shows the number of fixations on target-present trials split by target contrast (the number of fixations on target present and target-absent trials is shown in Fig. S13 (OSM)). These data were analysed with a three-way repeated-measures ANOVA with condition, repetition and target contrast as within-subject factors. One participant was excluded due to empty cells. The effect of cue was shown by the significant two-way interaction between condition and repetition. For the sake of completeness, we also report the other statistics. The effects of condition, repetition and target contrast were all significant [condition: F(1, 16) = 31.86, p < 0.001, ${\eta }_{p}^{2}$ = 0.666; repetition: F(1, 16) = 6.38, p = 0.022, ${\eta }_{p}^{2}$ = 0.285; target contrast: F(2, 32) = 32.78, p < 0.001, ${\eta }_{p}^{2}$ = 0.672]. All two-way interactions were significant [condition $\times$ repetition: F(1, 16) = 32.18, p < 0.001, ${\eta }_{p}^{2}$ = 0.668; condition $\times$ target contrast: F(2, 32) = 4.68, p = 0.016, ${\eta }_{p}^{2}$ = 0.226; repetition $\times$ target contrast: F(2, 32) = 10.33, p < 0.001, ${\eta }_{p}^{2}$ = 0.392]. The three-way interaction was not significant. Then three two-way repeated-measures ANOVAs with condition and repetition as within-subject factors were conducted for each target contrast separately.

Table 7 shows the results of the two-way ANOVA for each target contrast. When target contrast = 15, participants made fewer fixations on the second copy of trials, but the effect of condition and the interaction between condition and repetition were not significant, suggesting that the decrease in fixation number from the first to the second round was merely due to the effect of time on experiment (repetition or practice) rather than the cue. When target contrast = 45 or 75, the effect of repetition was not significant, but the effect of condition was. Participants made fewer fixations in the noCue – noCue condition than the noCue – Cue condition. The interaction between repetition and condition was also significant, with a larger effect of condition in round 2 (noCue vs. Cue) than in round 1 (noCue vs. noCue). This suggests that the increase in number of fixations from noCue – noCue to noCue – Cue was due to the presence of the cue.

**Table 7:** Results of the repeated-measures ANOVA on fixation number for each target contrast in Experiment 2 (Bonferroni-corrected α = 0.0167)

Target contrast	Effect	$F$	$p$	${\eta }_{p}^{2}$
15	Repetition	F(1, 16) = 12.713	0.003	0.443
	Condition	F(1, 16) = 1.462	0.244	0.084
	Repetition * condition	F(1, 16) = 0.263	0.615	0.016
45	Repetition	F(1, 16) = 0.315	0.583	0.019
	Condition	F(1, 16) = 58.442	< 0.001	0.785
	Repetition * condition	F(1, 16) = 16.148	< 0.001	0.502
75	Repetition	F(1, 16) = 0.687	0.419	0.041
	Condition	F(1, 16) = 16.665	< 0.001	0.510
	Repetition * condition	F(1, 16) = 18.122	< 0.001	0.531

The effect of cue on fixation number tracked what happened on RTs. The cueing did not significantly affect the number of fixations on low-contrast targets (target contrast = 15) but did increase the number of fixations when target contrast = 45 or 75. Therefore, the decrease in miss rates on cued low-contrast trials did not seem to be the result of an increase in the number of fixations on these trials.

##### *Fixation durations*

**Fig. 15:**
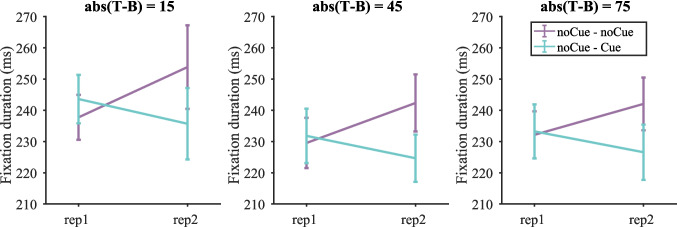
Fixation durations on target-present trials split by target contrast in Experiment 2. Error bars represent $\pm 1$ standard error

Figure 15 shows the fixation durations on target-present trials split by target contrast (fixation durations on target present and target-absent trials are shown in Fig. S14 (OSM)). Similar to the results in Experiment 1, the cue decreased fixation durations regardless of target contrast.

This was demonstrated by a three-way repeated measures ANOVA with condition, repetition and target contrast as within-subject factors. One participant was excluded due to empty cells. The effects of condition and target contrast were significant [condition: F(1, 16) = 23.58, p < 0.001, ${\eta }_{p}^{2}$ = 0.596; target contrast: F(2, 32) = 9.39, p < 0.001, ${\eta }_{p}^{2}$ = 0.370], but the effect of repetition was not [F(1, 16) = 0.855, p = 0.369, ${\eta }_{p}^{2}$ = 0.051]. Fixation durations were significantly longer with target contrast = 15 than with other high-contrast targets [15 vs. 45: t(16) = 5.08, p < 0.001; 15 vs. 75: t(16) = 2.81, p = 0.012]. The interaction between condition and repetition was significant F(1, 16) = 20.46, p < 0.001, ${\eta }_{p}^{2}$ = 0.561]. For the noCue – noCue condition fixation durations increased from repetition 1 to repetition 2, but for the noCue – Cue condition fixation durations actually decreased from repetition 1 to repetition 2. All other interactions were not significant. These results suggest that the decrease in fixation durations from noCue trials to Cue trials was not due to the effect of time in experiment (practice or repetition) but the presence of the cue.

##### *Saccade length*

**Fig. 16:**
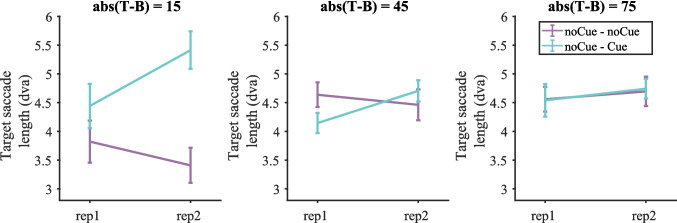
Target saccade length on target-present trials split by target contrast in Experiment 2. Error bars represent $\pm 1$ standard error

**Fig. 17:**
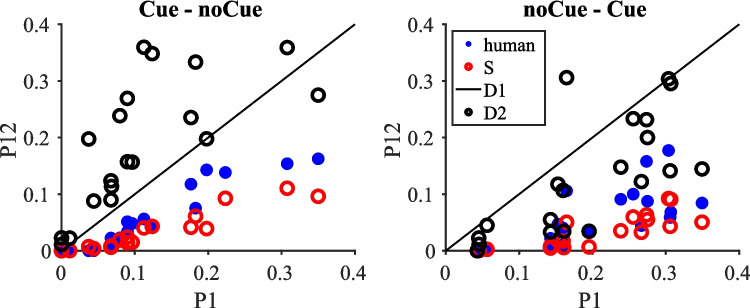
Comparison between human data and deterministic/stochastic predictions in Experiment 1. The stochastic prediction is $S = P1 * P2$. The deterministic predictions are $D1=P1$ and $D2 = P2$. (Please note that the D2 values lie above the line P1 = P12 for Cue-no Cue and below it for no Cue-Cue. This is due to the fact that the Cue condition yields fewer errors than the no Cue condition)

Figure 16 shows the average length of target saccades on target-present trials split by target contrast (Fig. S15 (OSM) shows target saccade length pooled over all target-present trials, Figure S16 (OSM) shows search saccade length on target present and target-absent trials, Figure S17 (OSM) shows search saccade length on target-present trials split by target contrast). Similar to the results in Experiment 1, the cue mainly increased target saccade length, which was demonstrated by a three-way repeated-measures ANOVAs with condition, repetition and target contrast as within-subject factors. One participant was excluded due to empty cells. The effect of condition was significant [F(1, 16) = 8.16, p = 0.011, ${\eta }_{p}^{2}$ = 0.338]. The effects of repetition and target contrast were almost significant [repetition: F(1, 16) = 3.30, p = 0.088, ${\eta }_{p}^{2}$ = 0.171; target contrast: F(1, 16) = 2.68, p = 0.084, ${\eta }_{p}^{2}$ = 0.143]. The two-way interaction between condition and repetition was significant [F(1, 16) = 6.05, p = 0.026, ${\eta }_{p}^{2}$ = 0.275]. The two-way interaction between condition and target contrast was also significant [F(2, 32) = 8.25, p = 0.001, ${\eta }_{p}^{2}$ = 0.340]. Other interactions were not significant. Then three two-way repeated-measures ANOVAs with condition and repetition as within-subject factors were conducted on target saccade length for each target contrast separately.

Table 8 shows the results of the two-way ANOVAs for each target contrast. When target contrast = 15, the effect of condition was significant, with larger saccades for noCue – Cue than for noCue – noCue. The interaction between condition and repetition failed to break the Bonferroni-corrected critical value of 0.0167, although the biggest difference in saccade length between the two conditions occurred on the second presentation, which does suggest that the larger saccades for noCue – Cue are caused by the cueing. When target contrast = 45, the effects of condition and repetition were not significant. The interaction between condition and repetition almost exactly meets the Bonferroni-corrected critical value of 0.0167 (p = 0.016706). The pattern is the same as for contrast = 15. The cueing seems to increase saccade length for the second presentation. When target contrast = 75, neither the effect of condition nor the effect of repetition was significant. The interaction between the two factors was not significant either.

**Table 8:** Results of the repeated-measures ANOVA on target saccade length for each target contrast in Experiment 2 (Bonferroni-corrected α = 0.0167)

Target contrast	Effect	$F$	$p$	${\eta }_{p}^{2}$
15	Repetition	F(1, 16) = 0.701	0.415	0.042
	Condition	F(1, 16) = 12.050	0.003	0.430
	Repetition * condition	F(1, 16) = 4.642	0.047	0.225
45	Repetition	F(1, 16) = 1.756	0.204	0.099
	Condition	F(1, 16) = 0.406	0.533	0.025
	Repetition * condition	F(1, 16) = 7.139	0.017	0.309
75	Repetition	F(1, 16) = 0.89	0.360	0.053
	Condition	F(1, 16) = 0.01	0.935	0.000
	Repetition * condition	F(1, 16) = 0.03	0.873	0.002

## Experiment 2: Discussion

**Fig. 18:**
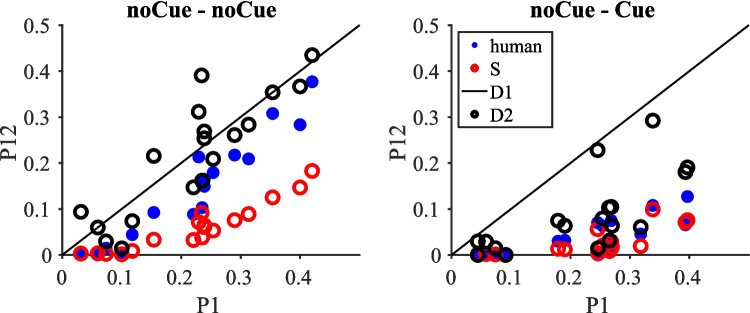
Comparison between human data and deterministic/stochastic predictions in Experiment 2. The stochastic prediction is $S = P1 * P2$. The deterministic predictions are $D1=P1$ and $D2 = P2$

**Fig. 19:**
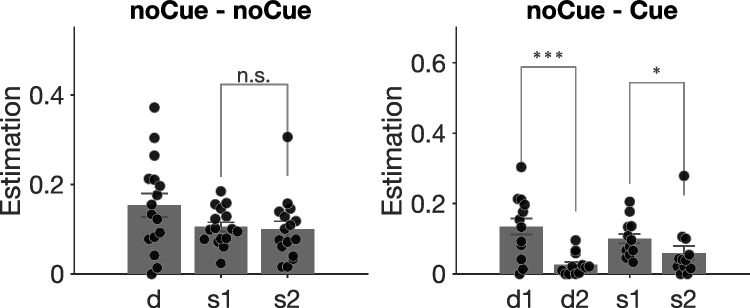
Estimation of deterministic error proportion and stochastic error rate for the noCue-noCue condition (**left**) and noCue-Cue condition (**right**). d: deterministic error rate in noCue-noCue; d1 deterministic error rate in round 1 of noCue-Cue; d2 deterministic error rate in round 2 of noCue-Cue; s1 stochastic error rate in round 1; s2 stochastic error rate in round 2

In Experiment 2, the stimuli were split into a noCue – noCue condition and a noCue – Cue condition. This was an attempt to more closely mirror the setup of our previous online experiment (Li et al., 2024). We found that the cue reduced miss errors but did not significantly affect RTs on low-contrast targets (target contrast = 15). However, on high-contrast targets (target contrast = 45 or 75), the cue did not reduce miss errors yet increased RTs. Similar to the results in Experiment 1, the cue also reduced the distance between distractors and closest fixation, especially for low-contrast items. For other eye-tracking measures, the cue did not significantly affect the number of fixations in trials with low-contrast targets, but did increase the number of fixations when target contrast was higher. Fixation durations were significantly reduced by the cue regardless of target contrast. Target saccade length was increased by the cue but search saccade length was decreased, though these effects were not robust. After the cueing intervention was implemented, both deterministic errors and stochastic errors were reduced (see Figs. 18 and 19 in the Appendix). Although this is different from the previous online Experiment 3c in Li et al. (2024) where only deterministic errors were reduced, it is still the case that most of the reduced errors were deterministic errors.

## General discussion

Errors in visual search are stubborn and potentially dangerous. Therefore, it is important to find ways to reduce errors and to understand the mechanisms underlying error reduction. In our previous work (Li et al., 2024), we found that an item-cueing intervention highlighting positions of all the letters reduced errors albeit at the cost of an increase in RT. It is important to recall that this cue does not preferentially highlight the target. It highlights *everything* that could be a target. In this paper, we further investigated the mechanisms of this cueing intervention with two eye-tracking experiments. Our observers were asked to search for the letter ‘T’ among the distractors ‘L’, with each stimulus presented twice. In Experiment 1, the stimuli were split into a Cue – noCue condition (where the cue was present only on the first copy of the stimuli) and a noCue – Cue condition (where the cue was present only on the second copy of the stimuli). In Experiment 2, the stimuli were split into a noCue – noCue condition (where the cue was present on neither the first copy nor the second copy) and a noCue – Cue condition (where the cue was present only on the second copy). Our main results are summarized in Table 9: (1) The cue reduced miss errors on low-contrast targets in both experiments. In principle, this outcome is compatible both with enhanced processing and more effective directing, since both would reduce the number of miss errors. (2) The cue did not significantly affect RTs regardless of target contrast in Experiment 1, but significantly increased RTs on high-contrast targets in Experiment 2; this outcome seems more compatible with more effective directing, since it suggests that some low contrast locations that were previously skipped were now visited. (3) In both experiments, the cue reduced the distance between distractors and the closest fixation, especially for low-contrast items; this outcome is also most compatible with more effective direction of attention, since it, again, indicates that low-contrast items that were previously skipped are now visited. (4) The cue did not significantly affect fixation number regardless of target contrast in Experiment 1, but significantly increased fixation number on high-contrast targets in Experiment 2. This outcome is again more compatible with more effective directing, since it hints at the fact that previously unvisited items are now fixated. (5) Fixation durations were significantly reduced by the cue in both experiments; this outcome is more compatible with enhanced processing, since it suggests that, in the presence of a cue, less time is needed to establish whether an item is a target or not. (6) In both experiments, target saccade length was increased by the cue while search saccade length was decreased by the cue, though these effects were not robust. This outcome is again more compatible with more effective direction, since the reduction in search saccade length indicates that fewer distractors were skipped. The increased target saccade length is probably due to the fact that participants made a saccade to the cue box surrounding the target, rather than the target itself.

**Table 9:** Cueing effects on behavioural and eye-tracking measures

Measures		Effect of cue in Experiment 1	Effect of cue in Experiment 2
Miss rate	Target contrast = 15	$\downarrow$	$\downarrow$
	Target contrast = 45	-	-
	Target contrast = 75	-	-
	Target contrast = 105	-	
RT	Target contrast = 15	-	-
	Target contrast = 45	-	$\uparrow$
	Target contrast = 75	-	$\uparrow$
	Target contrast = 105	-	
Fixation-item distance		$\downarrow$	$\downarrow$
Fixation number	Target contrast = 15	-	-
	Target contrast = 45	-	$\uparrow$
	Target contrast = 75	$\uparrow$	$\uparrow$
	Target contrast = 105	-	
Fixation duration	Target contrast = 15	$\downarrow$	$\downarrow$
	Target contrast = 45		
	Target contrast = 75		
	Target contrast = 105		
Target saccade length	Target contrast = 15	$\uparrow$(p = 0.017)	$\uparrow$(p = 0.047)
	Target contrast = 45	-	$\uparrow$(p = 0.017)
	Target contrast = 75	-	-
	Target contrast = 105	-	
Search saccade length	Target contrast = 15	$\downarrow$(p = 0.065)	$\downarrow$
	Target contrast = 45		
	Target contrast = 75		
	Target contrast = 105		

Taken together, these results support the interpretation that the cueing intervention primarily reduced miss errors because attention was more properly directed to the cued areas. However, it should be noted that the cue reduced positional uncertainty and allowed closer fixation of items. This will have improved foveation and may have allowed enhanced processing as well. (Although it should be noted that the largest reduction in miss rates happened for the low-contrast targets, suggesting that participants did not become that much better at recognizing higher contrast targets.) This main role for improved attentional allocation is consistent with other research revealing that a failure to inspect all the items is a leading cause of miss errors in visual search (Cain et al., 2013; Solman et al., 2014). Our findings suggest that simply enhancing an image in order to attract more attention to low-contrast item areas can be useful. Even if the enhancement is agnostic about the target and boosts the visibility of both targets and distractors (see Matzen et al., 2024).

Although the results were substantially consistent across the two eye-tracking experiments reported in this paper, it is worth noting that there were differences with our previous online experiments, even when we replicated the noCue – noCue versus noCue – Cue design in the eye-tracking Experiment 2. In our online Experiment 3c (Li et al., 2024), the cue slowed the search on both target present and target-absent trials. However, in eye-tracking Experiment 2, it only slowed search on trials with high-contrast targets. Therefore, we further compared both d’ and RTs from Li et al.’s (2024) online Experiment 3c and the current eye-tracking Experiment 2. The target contrast (abs(T − B)) in online Experiment 3c ranged from [15, 45, 75, 105] while the target contrast in the current Experiment 2 ranged from [15, 45, 75]. To make a fair comparison, all of the following analyses excluded target-present trials with target contrast = 105 from the online data. Figure S18 (OSM) shows the comparison of d’. The d’ from online participants was significantly lower than that from eye-tracking participants. Figure S19 (OSM) shows the comparison of RTs. On target-absent trials, eye-tracking participants generally searched longer while online participants seemed to quit much earlier. On target-present trials, eye-tracking participants found targets a bit faster. To sum up, online participants missed more targets, spent more time in finding targets and quit earlier on target-absent trials, suggesting that online participants probably put less effort into the task than eye-tracking participants. The potential reasons for these differences include, but are not limited to: (1) The minimum distance between every two letters was larger in the eye-tracking experiment (0.12 screen height) than in the online experiment (0.1 screen height), so crowding effects in the online experiment were probably larger. (2) The physical screen height was larger in the eye-tracking experiment (27 cm) than in the online experiment (average $\approx$ 23.7 cm). Since the stimuli fully occupied screen height in both experiments, the physical size of the stimulus will have been smaller for our online participants. (3) There were more blocks in the eye-tracking experiment (12 blocks) than in the online experiment (four blocks). Participants got more chances to take a break and were given feedback (block accuracy) more often in the eye-tracking experiment. (4) There were some individual differences between online participants recruited from Prolific and eye-tracking participants recruited from the BSc Psychology programme at the University of Manchester. (5) The presence of an experimenter. Whereas the online participants did the experiment unsupervised in an environment of their choosing, the eye-tracking participants shared the lab with the experimenter without any other distractions. This may have encouraged and enabled them to do their best. The differences we found between our online experiment and our in-person experiments highlight that the two modes of experimentation might not always be directly comparable.

Given that one of our inspirations for this work is the need for the reduction of miss errors in radiography, it is important to discuss what our results mean for real-world applications. Here, there are two important considerations: effectiveness (i.e., make as few miss errors as possible) and efficiency (take as little time as possible to achieve this).

In both experiments we found that highlighting all potential targets reduced miss errors on low-contrast targets without a concomitant increase in RTs. However, in Experiment 2 we did find an increase in the RTs for higher contrast targets. This suggests that although cueing improves the detection of difficult to find targets, it also creates a drag on easier to find targets. Both seem to be two sides of the same coin: the cue box guides attention to low-contrast items. If the item is a target, a miss error is prevented (increased effectiveness), but if the item is a distractor it will take up valuable time to establish this fact (reduced efficiency). Part of this may be a novelty effect: in Experiment 1, where 50% of the displays were cued (rather than the 25% in Experiment 2), the detrimental effect on RTs was muted.

For real-world applications this result holds out hope: there seems to be a sweet spot where it is possible to increase effectiveness without decreasing efficiency.

In conclusion, in this work we used eye tracking to establish how the errors in visual search can be reduced by a cueing intervention that highlights the positions of all potential targets. Eye-tracking data demonstrated that when the cueing intervention was present, observers fixated closer to items with shorter fixation durations. The smaller fixation-item distance resulting from the cueing reduced low-contrast search errors. The remaining low-contrast errors seemed to be recognition errors. These results suggest that the main reason that errors were reduced in our experiments was because attention was more properly directed to the cued areas instead of being enhanced at the cued areas. Additionally, there may have been some role for enhanced attentional processing due to the better foveation of items that cueing their position allowed. Therefore, in visual search tasks with low-contrast targets, interventions like image-enhancing algorithms could be effective in properly directing attention to low-contrast areas and thus reducing miss errors. Importantly, this reduction in miss rates will not necessarily have to come at a cost to RTs.

## Electronic supplementary material

Below is the link to the electronic supplementary material.

Supplementary file1 (DOCX 2943 kb)

## Data Availability

Data and materials are available at request to the corresponding author (JH).

## Appendix 1

### Experiment 1: Stochastic errors and deterministic errors

Following up on our previous work, we can assess the relative contributions of stochastic and deterministic processes to the errors in this experiment. The classification of errors as stochastic or deterministic was based on the miss rate on the first copy of trials $P1$, the miss rate on the second copy of trials $P2$, and the miss rate on both copies $P12$. The stochastic prediction, assuming complete independence of errors on first and second copies, is $S = P1 * P2$. This means that all errors are stochastic errors if $P12 = S$. The deterministic predictions^*^ are $D1=P1$ and $D2 = P2$ which means that all errors in round 1 (or round 2) are deterministic if $P12 = D1$ (or $P12 = D2$). Figure 17 shows that in both conditions, the errors were a mix of stochastic and deterministic errors.

In principle, the deterministic error proportions (relative to the total number of stimuli) $d1$, $d2$ as well stochastic error rates (for one stimulus) $s1$, $s2$ can be derived from $P1$, $P2$ and $P12$ as in our previous work (Li et al., 2024). However, in this experiment, only $s1$, $d1$ in the Cue – noCue condition and $s2$, $d2$ in the noCue – Cue condition can be uniquely determined (due to the fact that the cue has an influence on the error rates, it is not possible to use the uniquely determined values from the Cue trials to estimate the values from the noCue trials). Since both estimated $s$ and $d$ are supposed to be positive rates (including 0), any participants with estimates smaller than -0.002 were excluded and any estimate between – 0.002 and 0 was rounded to 0. Two participants were excluded in the Cue – noCue condition and two participants were excluded in the noCue – Cue condition. We could not calculate whether deterministic errors or stochastic errors were reduced in this experiment, but we found that both $d1$ (mean = 0.0413, min = 0, max = 0.1388) in the Cue – noCue condition and $d2$ (mean = 0.0452, min = 0, max = 0.1491) in the noCue – Cue condition were significantly different from 0 [$d1$ in Cue—noCue: t(17) = 3.74, p = 0.002, Cohen’s d = 0.88; $d2$ in noCue—Cue: t(17) = 4.19, p < 0.001, Cohen’s d = 0.99], suggesting that the cueing intervention did not completely eliminate deterministic errors.

^*^ In principle, the deterministic prediction is D1 = P1 = P2 = D2: Every miss on the first copy will be repeated on the second copy. However, since one of the repetitions has a cue which is known to reduce miss errors the condition P1 = P2 is unlikely to hold.

### Experiment 2: Stochastic errors and deterministic errors

Figure 18 shows that the errors are a mix of stochastic and deterministic errors in the noCue – noCue condition but seem to shift more towards stochastic in the noCue – Cue condition.

The deterministic error proportions (relative to the total number of stimuli) $d1$, $d2$ as well stochastic rates (for one stimulus) $s1$, $s2$ were derived from $P1$, $P2$ and $P12$ as in our previous work (Li et al., 2024). Since estimated $s1$, $s2$ and $d1$, $d2$ are supposed to be positive rates (including 0), any participants with estimations smaller than -0.002 were excluded and any estimation between – 0.002 and 0 was rounded to 0. Figure 19 shows the estimated parameters in Experiment 2. In the noCue – noCue condition, two participants were excluded. The difference between $s1$ and $s2$ was not significant. (Please note that there is only a single estimate for the deterministic rate d here, since we assume that the deterministic rate does not change from round 1 to round 2 for noCue – noCue. This estimated d was subsequently used as the estimate for d1 in the noCue – Cue condition.) In the noCue – Cue condition, six participants were excluded. Paired t-tests suggest $s2$ was significantly smaller than $s1$ [t(11) = 2.26, p = 0.045, Cohen’s d = 0.65], and $d2$ was significantly smaller than $d1$ [t(11) = 5.10, p < 0.001, Cohen’s d = 1.47]. $d2$ was still significantly larger than 0 [t(11) = 3.00, p = 0.012, Cohen’s d = 0.87], suggesting that the cueing intervention reduced but did not eliminate deterministic errors.

## Footnotes

1 This definition is different from the one used in Wu et al. (2022). Compared with the region of interest (ROI)-based definition used in their work, it has the advantage of not having to set a single ROI size. A single ROI size is hard to justify in the current experiments, because the contrast between items and background varied greatly. However, there are also some limitations with regard to this definition: (1) The closest fixation might be more than 10 degrees from the target, and it is unclear whether such fixations are meaningful as target fixations; (2) if the last five fixations and their distances to target are *A: 5 deg*, *B: 1 deg*, *C:4 deg*, *D: 0.5 deg*, *E: 5 deg*, our definition will make the fixation *D* with distance *0.5 deg* the target fixation, but both fixation *B* with distance *1 deg* and fixation *D* with distance *0.5 deg* may be the real target fixation. Although these two limitations will affect the selection of target fixation to some extent, they will not substantially affect our main conclusions related to the definition of target fixation. First, the blue lines in the bottom rows of Figs. 5 and 12 show the distributions of distance between the target fixation and the target, which demonstrate that a large proportion of distance values fell within 2 degrees and almost all distance values fell within 4 degrees. This suggests that very few target fixations were as far away from the target as indicated by the first limitation. Second, the second limitation might lead to the result that some post-target saccades were misclassified as target saccades, but such potential misclassification will occur for both Cue and noCue trials. Therefore, it is unlikely that our conclusions will be substantially affected by the misclassification. That said, it is still worth determining the best ROI size for the target fixation by a set of more sophisticated experiments designed for this question in the future.

2 The fixation-item distance was defined as the Euclidean distance between the fixation location and the centre of the item (sqrt((X_centre_-X_fixation_)^2^ + (Y_centre_-Y_fixation_) ^2^). For Figs. 6 and 13, we computed the distance between any fixation and any item and selected the smallest distance. Since participants have a tendency to fixate the target once they have found it, we only used distractor items in this computation.

## Code availability

Relevant code will be made available at request to the corresponding author (JH).

## Declarations

### Open practices statement

Data are available at request to the corresponding author (JH). None of the experiments was preregistered.
