# Supplementary material for: Errors in visual search: How can we reduce them?
Source: Atten Percept Psychophys. 2025 Jun 13;87(5):1471–95. doi: 10.3758/s13414-025-03095-6 (PMC12205024; doi:10.3758/s13414-025-03095-6)

# Supplementary material

Figure S1 shows miss rate data on target present trials in Experiment1. The orange dots representing the Cue – noCue condition are mostly clustered above the diagonal line of $P1=P2$ while the green dots representing the noCue – Cue condition are mostly below the diagonal line. This clearly indicates that the presence of the cue reduces the miss rates. (In the first presentation for Cue – noCue [P1<P2] and in the second presentation for noCue – Cue [P2>P1]


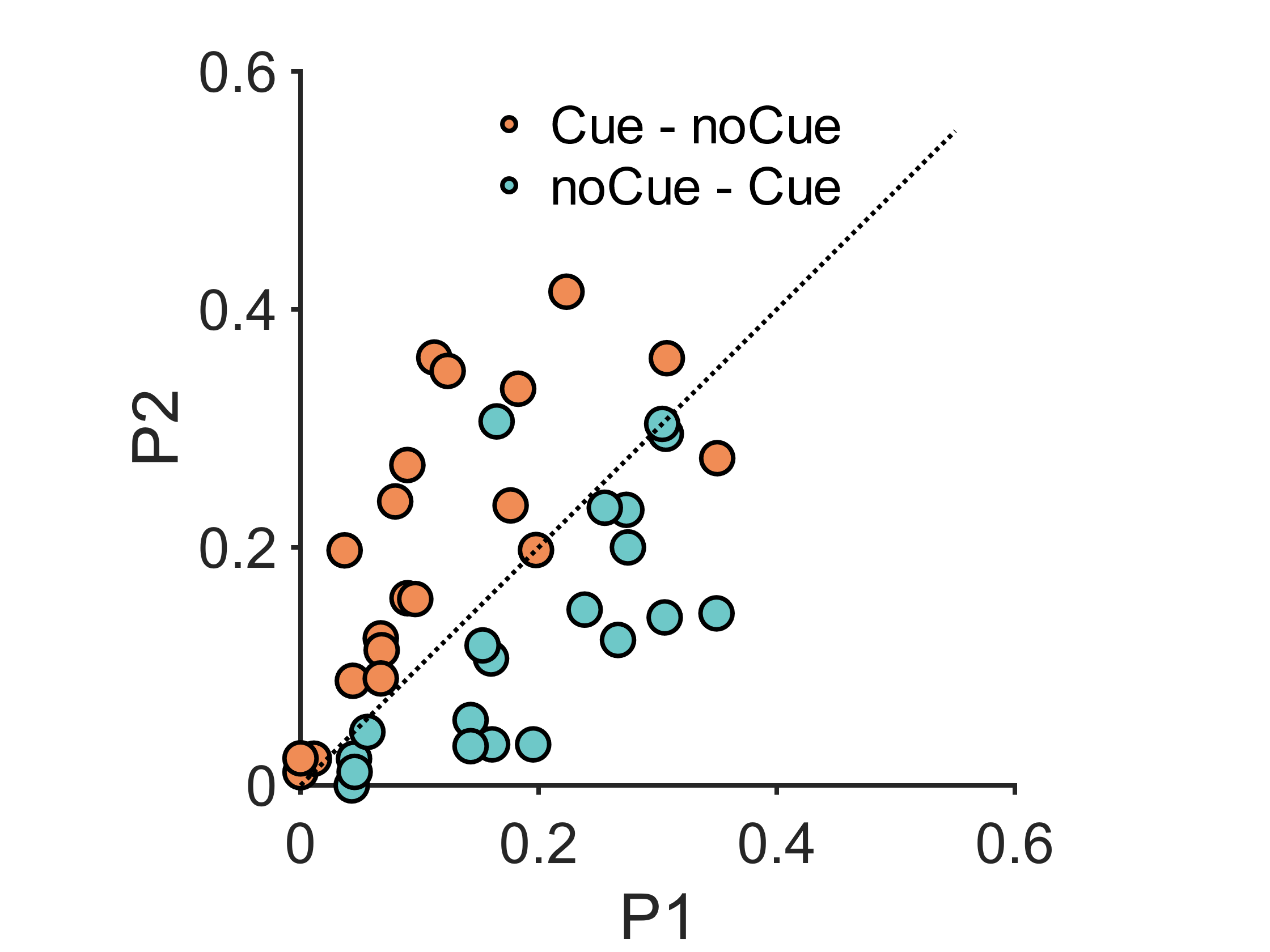


Figure S1. Miss rates on target present trials in Experiment 1.

Figure S2 shows the overall true positive and true negative RTs in Experiment 1. A three-way repeated measures ANOVA with target presence, condition and repetition as within-subject factors was conducted on the RTs. Since the experiment involved a typical visual search task, we found the usual main effect of target presence, which is statistically highly reliable and will not be reported in detail. The effect of repetition was significant [F(1, 19) = 5.20, p = 0.034, $\eta_{p}^{2}$ = 0.215], but the effect of condition was not [F(1, 19) = 0.001, p = 0.970, $\eta_{p}^{2}$ = 0.000]. The interaction between repetition and condition was not significant either [F(1, 19) = 1.50, p = 0.235, $\eta_{p}^{2}$ = 0.073], suggesting that the cue did not affect the RTs. None of the other two-way interactions were significant [target presence $\times$ repetition: F(1, 19) = 2.61, p = 0.123, $\eta_{p}^{2}$ = 0.121; target presence $\times$ condition: F(1, 19) = 0.018, p = 0.894, $\eta_{p}^{2}$ = 0.001]. The three-way interaction between the three factors came close [F(1, 19) = 4.28, p = 0.053, $\eta_{p}^{2}$ = 0.184]. The latter seems to be carried by the absent trials, where Cue was faster than NoCue for both conditions.


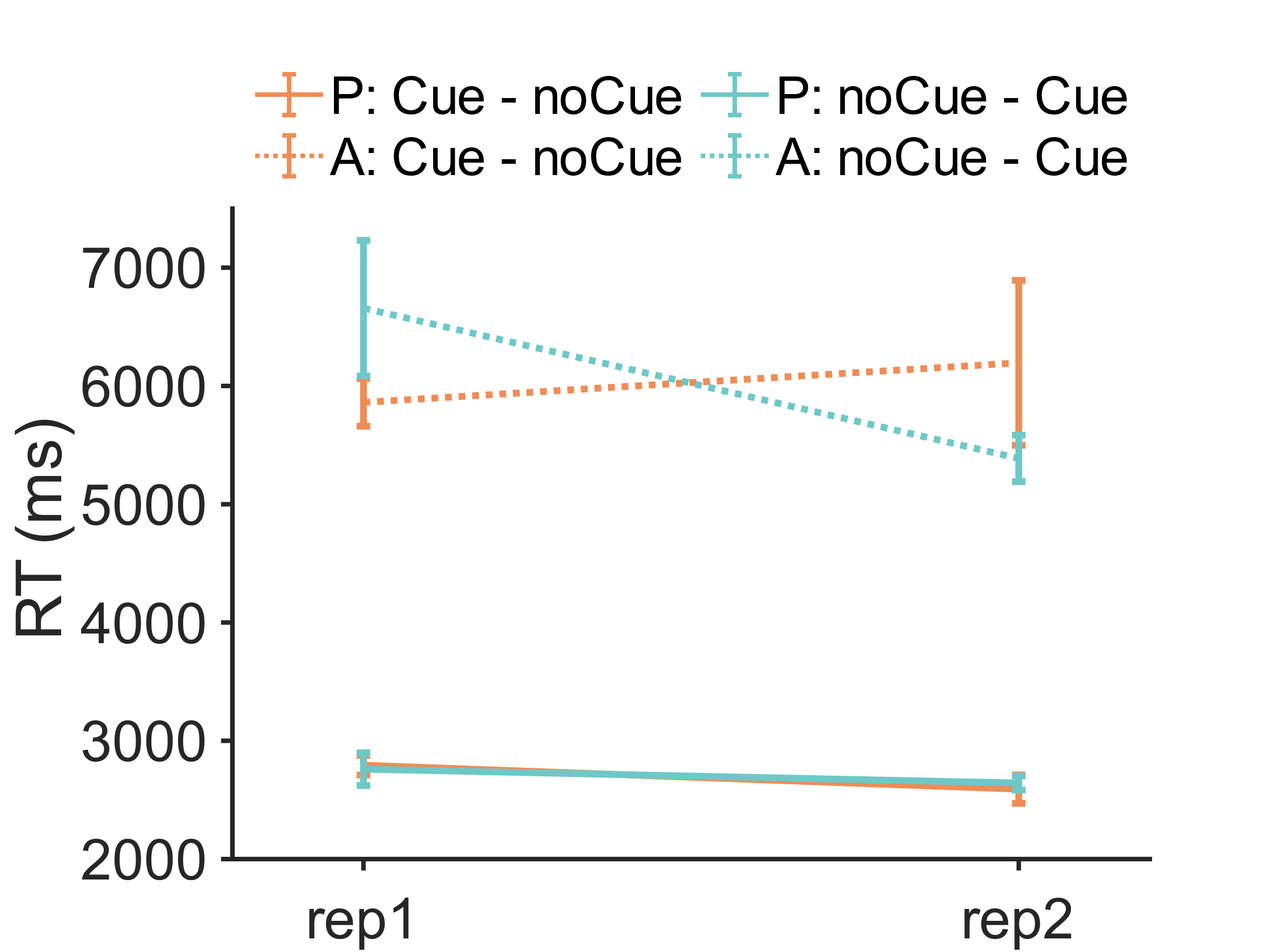


Figure S2. RTs on target present (solid line) and target absent (dashed line) trials in Experiment 1. Error bars represent $\pm1$ standard error.


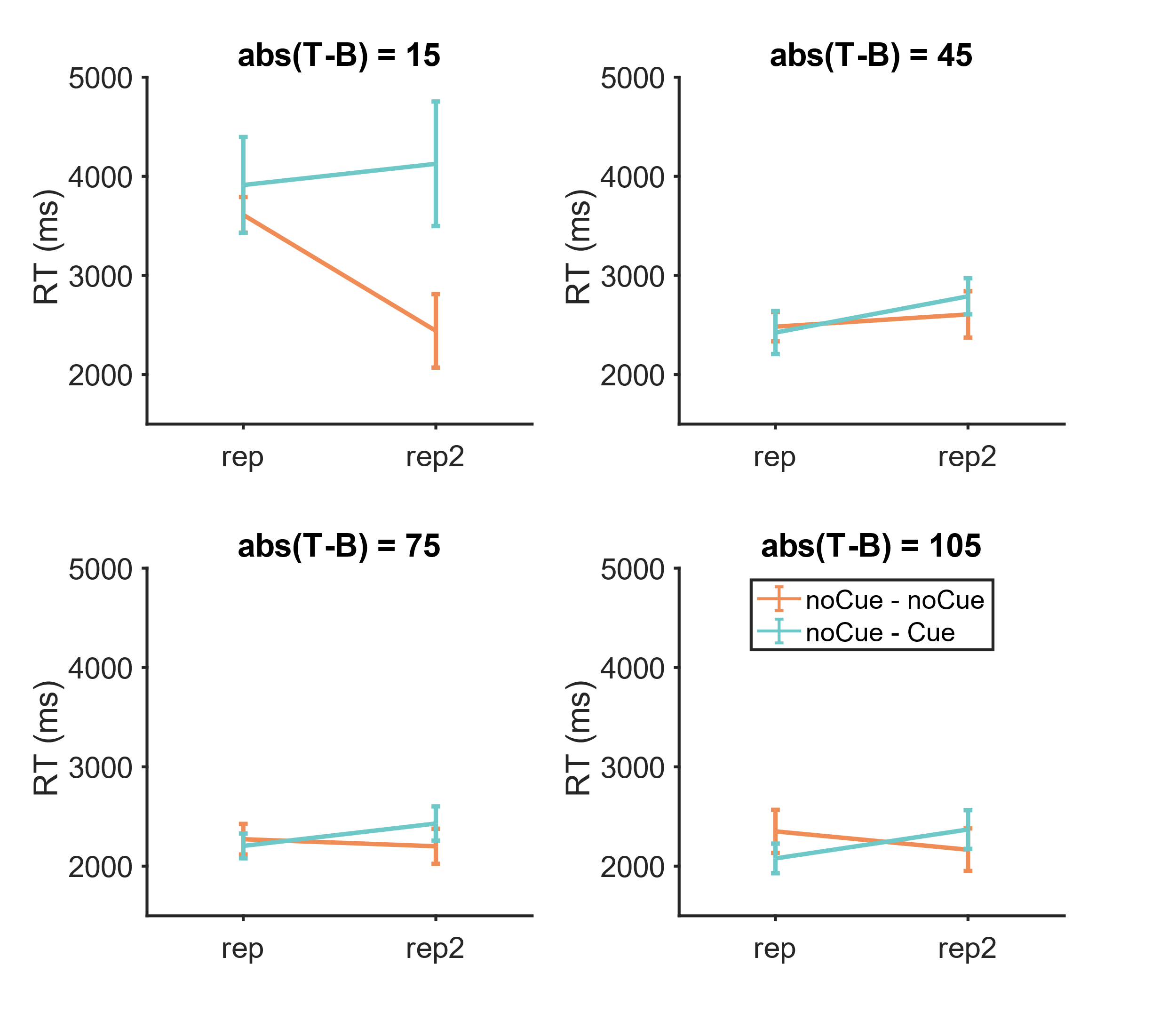


Figure S3. RTs on target present trials split by target contrast in the previous online experiment (Li et al., 2024).

Figure S4 shows the probability of the next fixation being on the target as a function of fixation-target distance in Experiment 1. This was a bit different from the Figures 7B and 7C in Wu et al. (2022), where there was a monotonic trend. This might be due to the differences in the definition of target fixation. Wu et al. set an ROI of 1.5 deg and chose the first fixation that fell into the ROI within five fixations of the end of the trial, while we chose the closest fixation within five fixations at the end the trial after eliminating the correction saccades. Therefore, in an example of target-fixation distance sequence [4.67, 3.82, 8.17, 7.56, 5.93, 5.60, 8.64, 13.99, 3.89,4.18, 1.03, 2.51, 1.06, 0.96], Wu et al. would have the fixation with distance 1.03 as the target fixation while we would have the fixation with distance 1.06 as the target fixation (0.96 was the closest but its distance from the previous fixation was smaller than 1 deg, so the previous fixation with 1.06 would be the target fixation). That is probably why our data has the bin of 1 deg in Figure S4 that led to a non-monotonic trend. If we focus on the data starting from the bin of 2 deg as Wu et al (2022) showed in their paper, Figure S4 shows the same monotonic trend just as the results in Wu et al. (2022).


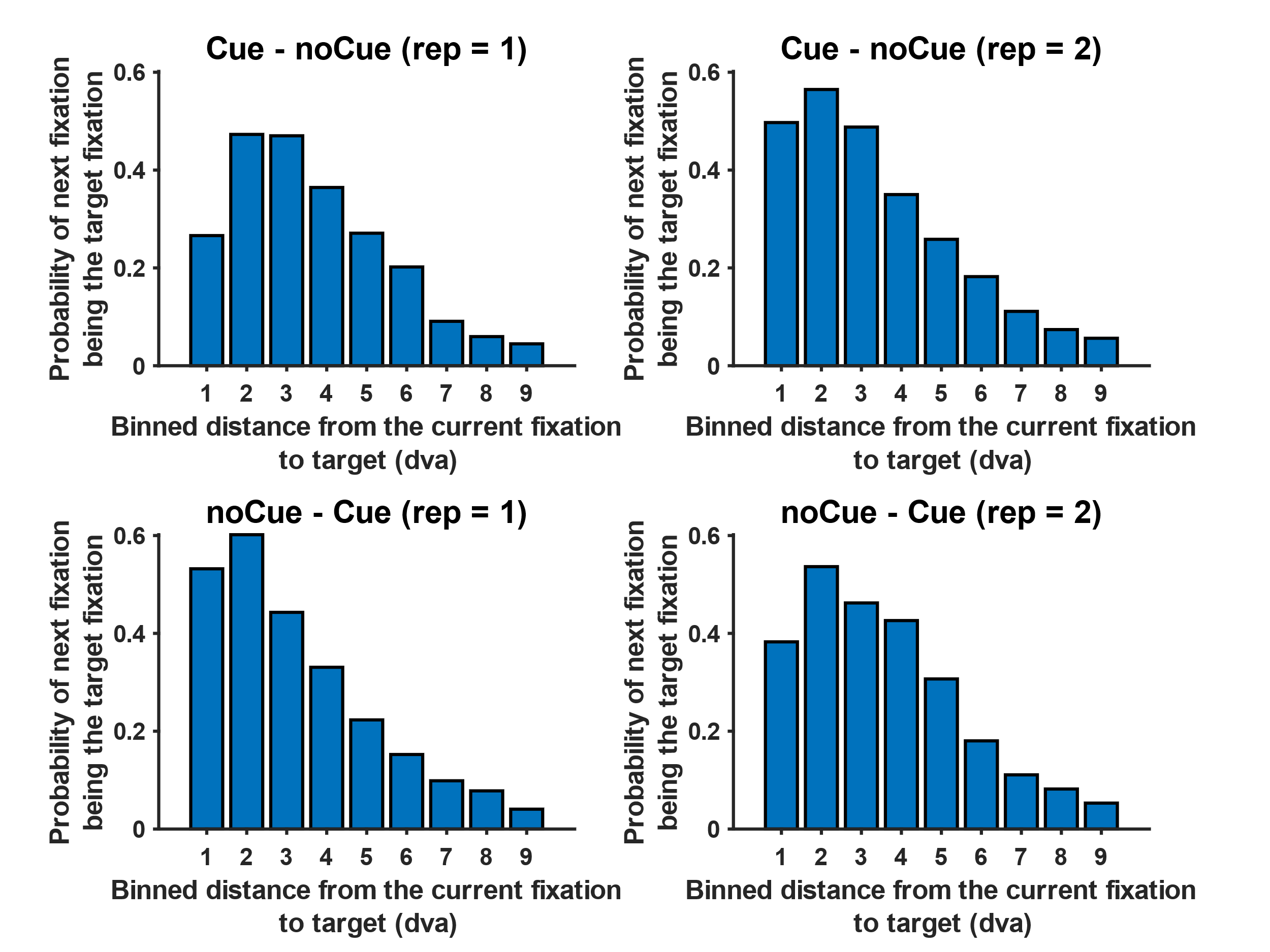


Figure S4. Probability of the next fixation being on the target as a function of fixation-target distance in Experiment 1.

Figure S5 shows the number of fixations in Experiment 1. A three-way repeated measures ANOVA with target presence, condition and repetition as within-subject factors was conducted on fixation number. The effects of target presence and repetition were significant [target presence: F(1, 19) = 119.60, p < 0.001, $\eta_{p}^{2}$ = 0.863; repetition: F(1, 19) = 8.52, p = 0.009, $\eta_{p}^{2}$ = 0.310], but the effect of condition was not [F(1, 19) = 0.11, p = 0.739, $\eta_{p}^{2}$ = 0.006]. None of the two-way interactions were significant [condition $\times$ target presence: F(1, 19) = 0.015, p = 0.903, $\eta_{p}^{2}$ = 0.001; condition $\times$ repetition: F(1, 19) = 0.020, p = 0.888, $\eta_{p}^{2}$ = 0.001; target presence $\times$ repetition: F(1, 19) = 3.60, p = 0.073, $\eta_{p}^{2}$ = 0.159]. The three-way interaction was not significant either [F(1, 19) = 1.83, p = 0.192, $\eta_{p}^{2}$ = 0.088]. Qualitatively, the number of fixations is congruent with the RTs in Figure S2.


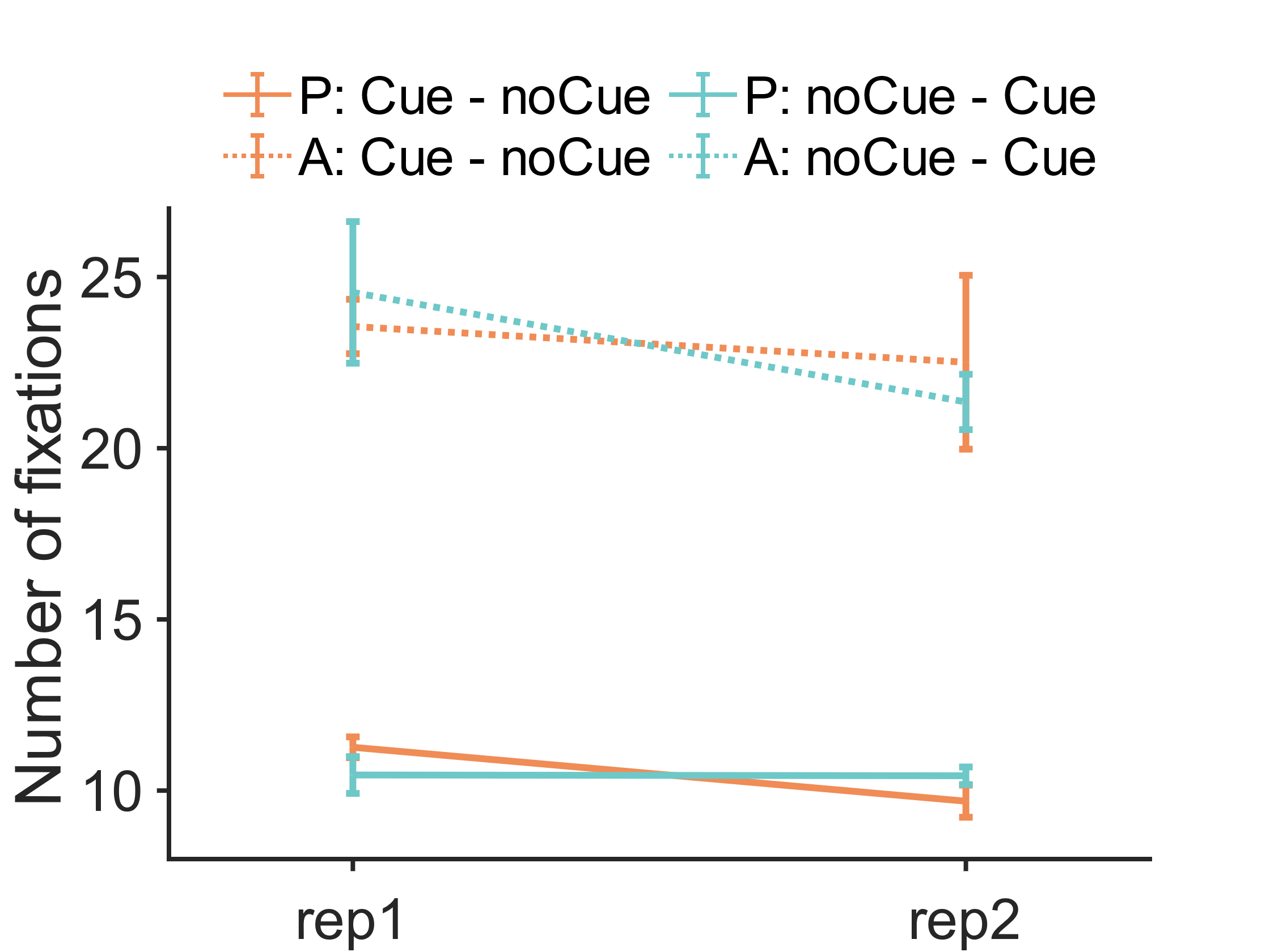


Figure S5. Number of fixations on target present (solid line) and target absent (dashed line) trials in Experiment 1. Error bars represent $\pm1$ standard error.

Figure S6 shows the fixation durations in Experiment 1. A three-way repeated measures ANOVA with target presence, condition and repetition as within-subject factors was conducted. The effects of target presence and repetition were significant [target presence: F(1, 19) = 99.82, p < 0.001, $\eta_{p}^{2}$ = 0.840; repetition: F(1, 19) = 5.89, p = 0.025, $\eta_{p}^{2}$ = 0.237], but the effect of condition was not [F(1, 19) = 0.18, p = 0.679, $\eta_{p}^{2}$ = 0.09]. The two-way interaction between repetition and condition was significant [F(1, 19) = 80.61, p < 0.001, $\eta_{p}^{2}$ = 0.809]. The three-way interaction between repetition, condition and target presence was also significant [F(1, 19) = 9.36, p = 0.006, $\eta_{p}^{2}$ = 0.330]. Therefore, two two-way repeated measures ANOVAs were conducted for target present and target absent trials separately. For target present trials, the interaction between condition and repetition was significant [F(1, 19) = 46.90, p < 0.001, $\eta_{p}^{2}$ = 0.712]. The effect of condition was not significant [F(1, 19) = 0.442, p = 0.514, $\eta_{p}^{2}$ = 0.023]. The effect of repetition failed to break the Bonferroni corrected critical value of .025 [F(1, 19) = 5.17, p = 0.035, $\eta_{p}^{2}$ = 0.214]. For target absent trials, the interaction between condition and repetition was significant [F(1, 19) = 70.71, p < 0.001, $\eta_{p}^{2}$ = 0.788]. The effects of condition and repetition were not significant [condition: F(1, 19) = 0.201, p = 0.659, $\eta_{p}^{2}$ = 0.010; repetition: F(1, 19) = 3.65, p = 0.071, $\eta_{p}^{2}$ = 0.161]. So, both for target present and target absent trials, fixation durations were shorter for the Cue condition than for the noCue condition.


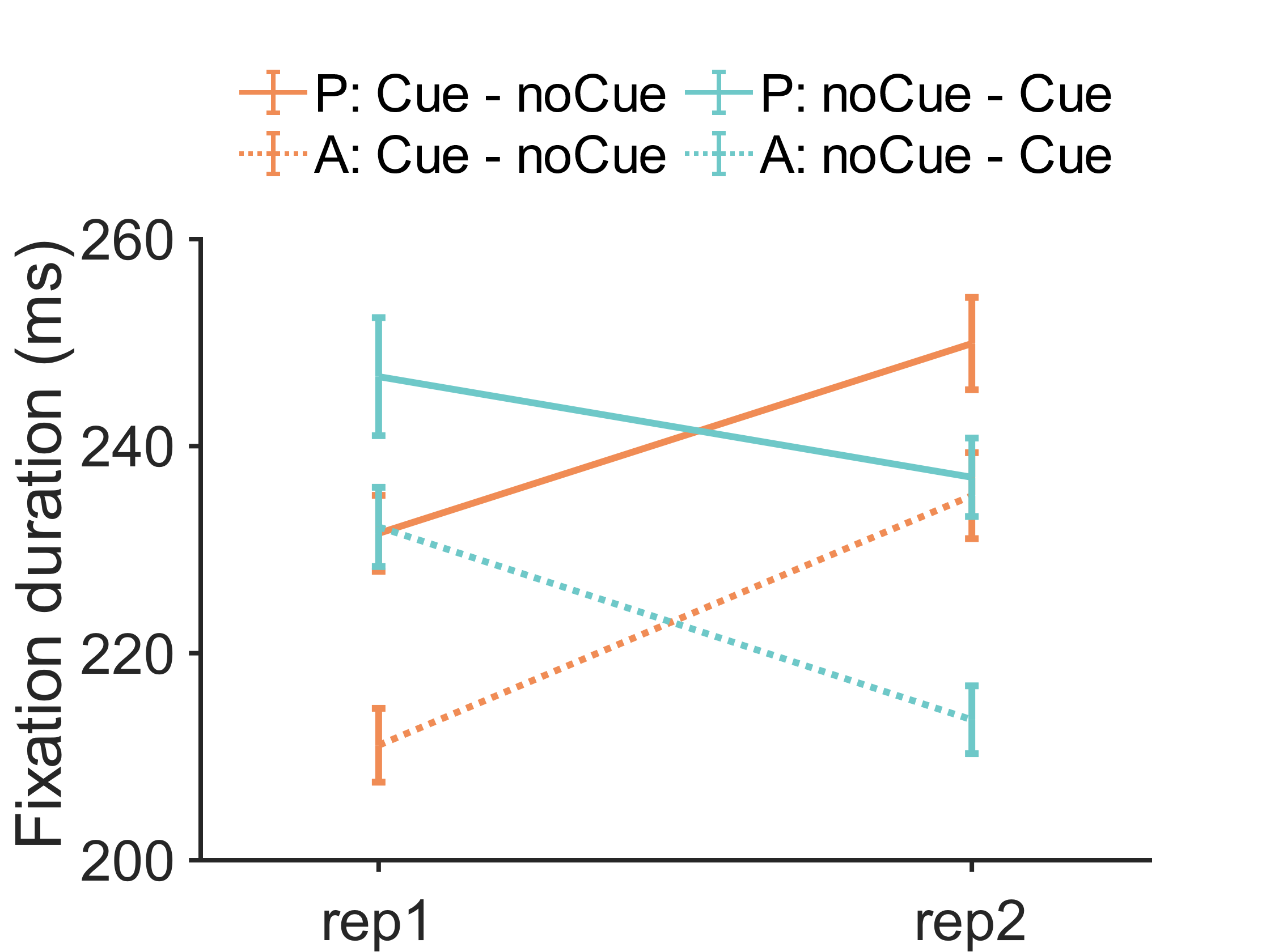


Figure S6. Fixation durations on target present (solid line) and target absent (dashed line) trials in Experiment 1. Error bars represent $\pm1$ standard error.

Figure S7 shows the average length of target saccade on target present trials in Experiment 1.


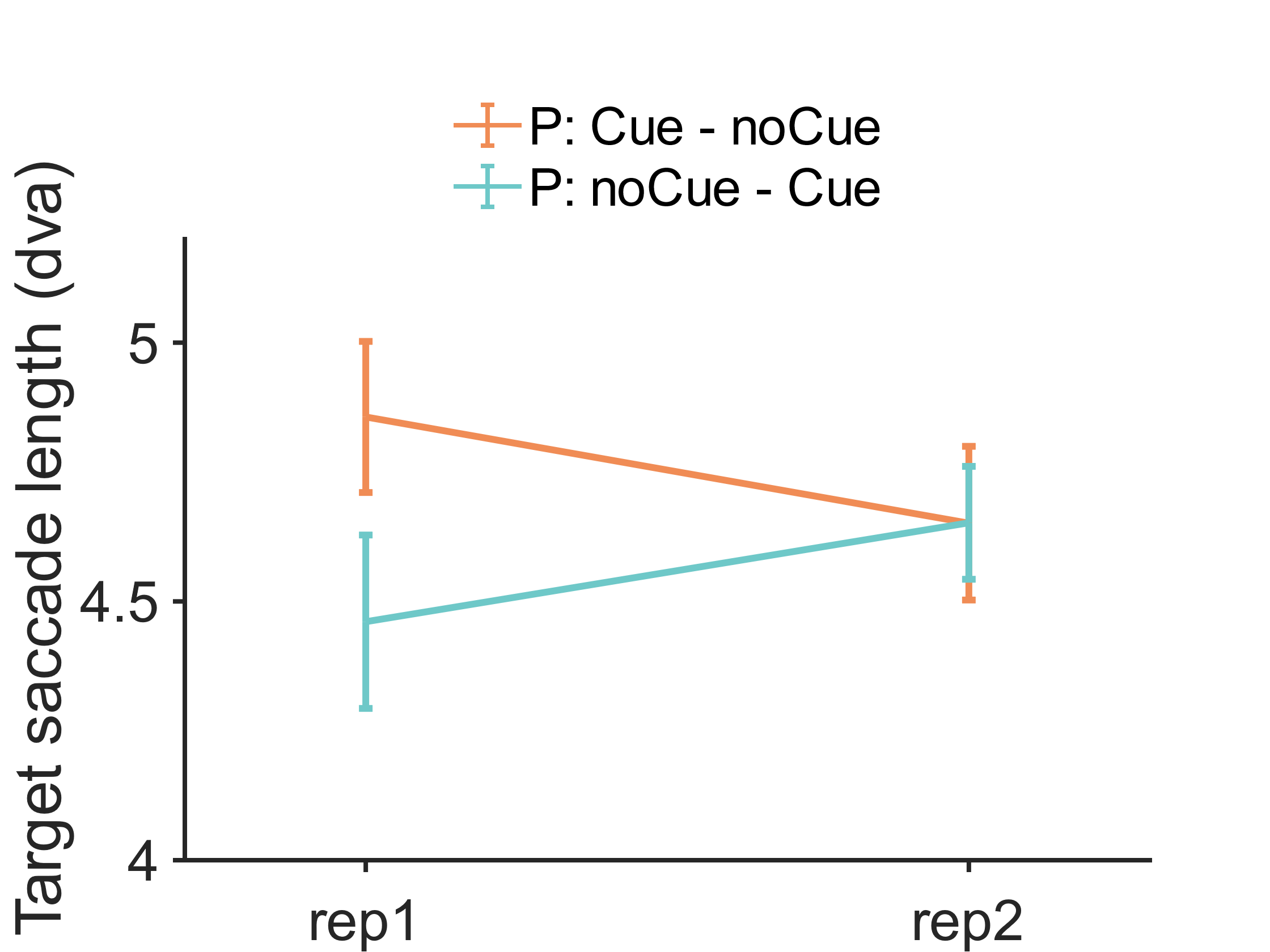


Figure S7. Target saccade length on target present trials in Experiment 1. Error bars represent $\pm1$ standard error.

Figure S8 shows the average length of search saccades in Experiment 1. For search saccade length, a three-way repeated measures ANOVA with cue, target presence and repetition as within-subject factors was conducted. The effect of target presence was significant [F(1, 19) = 60.54, p < 0.001, $\eta_{p}^{2}$ = 0.761], but the effects of repetition and condition were not [repetition: F(1, 19) = 0.06, p = 0.816, $\eta_{p}^{2}$ = 0.003; condition: F(1, 19) = 0.00, p = 0.979, $\eta_{p}^{2}$ = 0.000]. The interaction between condition and repetition was almost significant [F(1, 19) = 3.84, p = 0.065, $\eta_{p}^{2}$ = 0.168]. This reflected a trend towards shorter search saccades for the Cue condition. The other two-way interactions and the three-way interaction were not significant.


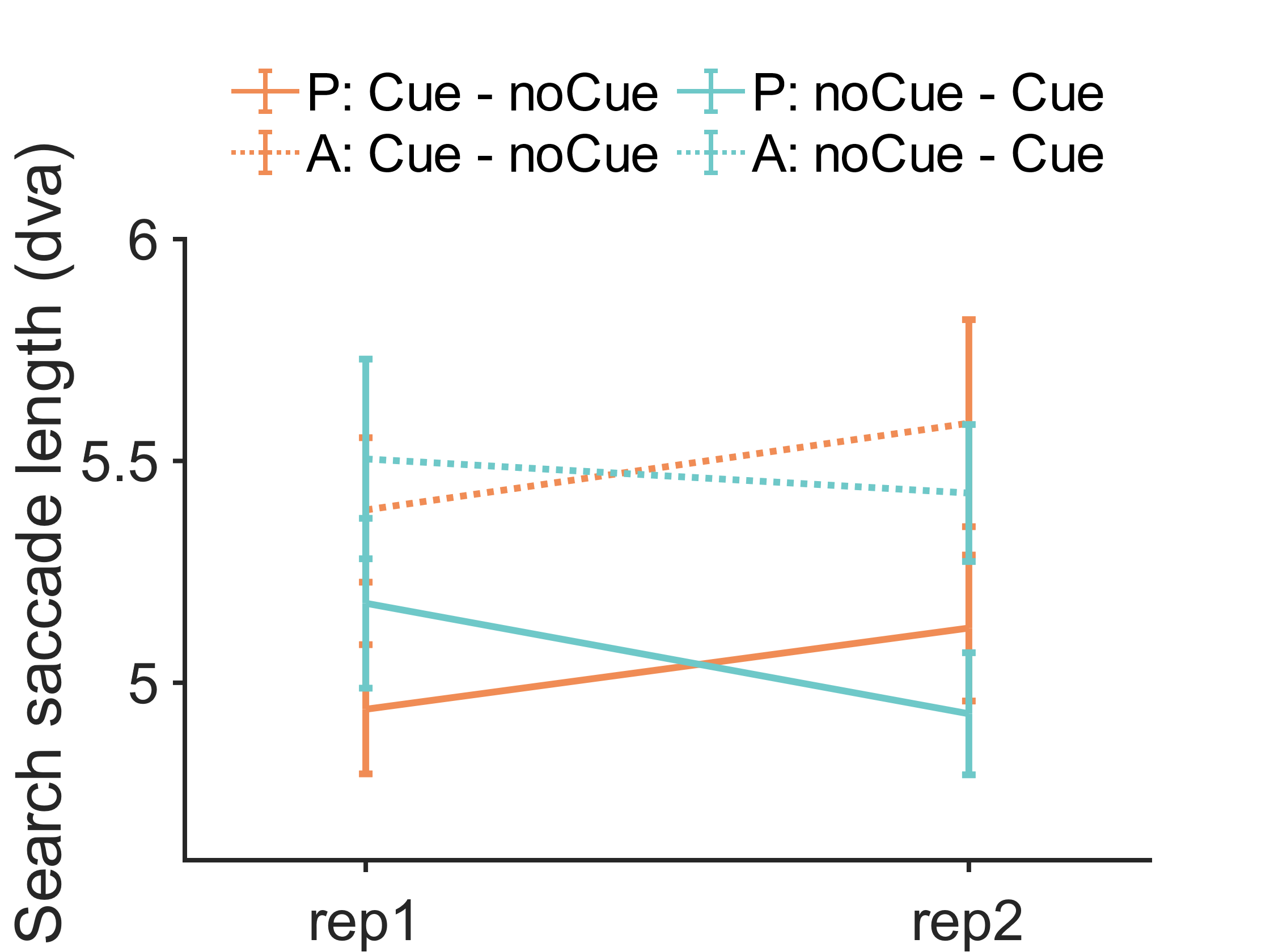


Figure S8. Search saccade length on target present (solid line) and target absent (dashed line) trials in Experiment 1. Error bars represent $\pm1$ standard error.

Figure S9 shows search saccade length on target present trials split by target contrast. A three-way repeated measures ANOVA with condition, repetition and target contrast as within-subject factors were conducted on search saccade length. Four participants were excluded due to empty cells. There was a trend towards shorter search saccades on Cue trials, but all effects and interactions were non-significant.


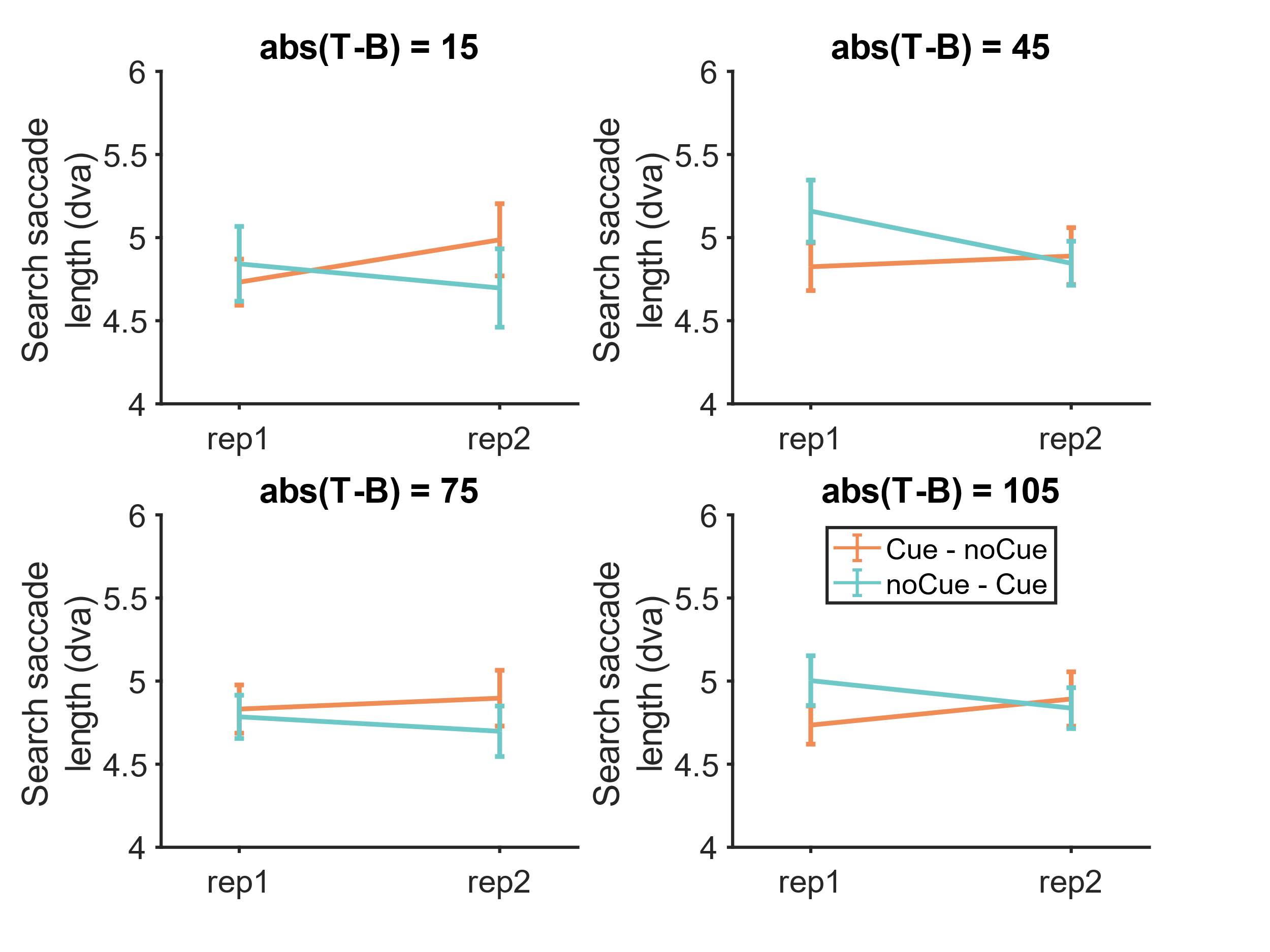
 Figure S9. Search saccade length on target present trials split by target contrast in Experiment 1. Error bars represent $\pm1$ standard error.

Figure S10 shows miss rate data on target present trials in Experiment 2. The purple dots representing the noCue – noCue condition are clustered around the diagonal line of $P1=P2$ while the green dots representing the noCue – Cue condition are mostly below the diagonal line, replicating the previous result that miss rates are reduced at the presence of the cue.


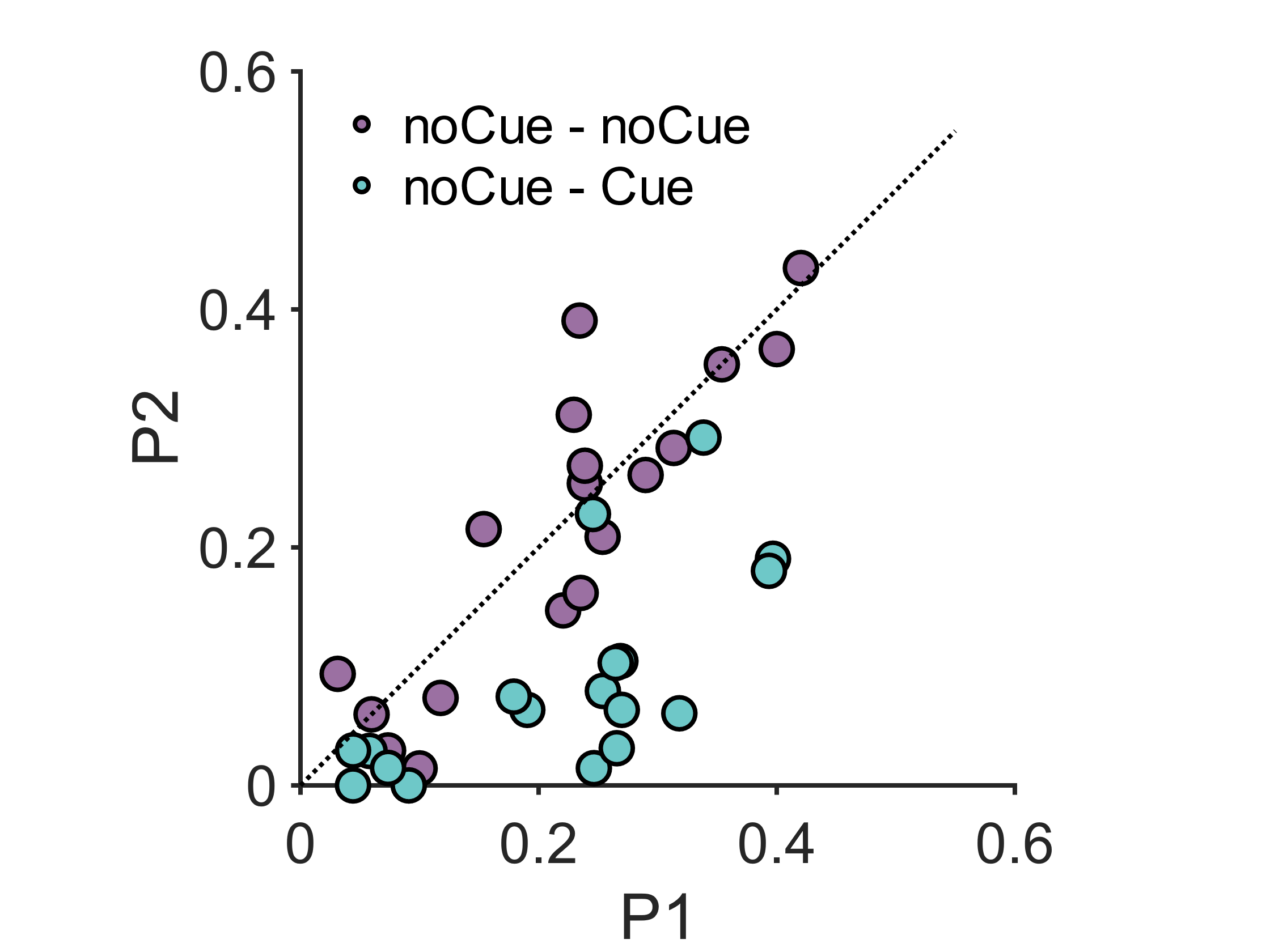


Figure S10. Miss rates on target present trials in Experiment 2.

Figure S11 shows RTs in Experiment 2. A three-way repeated measures ANOVA with target presence, condition and repetition as within-subject factors was conducted on RTs. The effect of repetition was significant [F(1, 17) = 9.00, p = 0.008, $\eta_{p}^{2}$ = 0.346], but the effect of condition was not significant [F(1, 17) = 1.16, p = 0.297, $\eta_{p}^{2}$ = 0.064]. The interaction between target presence and repetition was significant [F(1, 17) = 10.58, p = 0.005, $\eta_{p}^{2}$ = 0.384], indicating that the effect of repetition was larger on target absent trials. All other two-way interactions and the three-way interaction were not significant. Most importantly, there is again no evidence that the presence of the cue impedes reaction times.


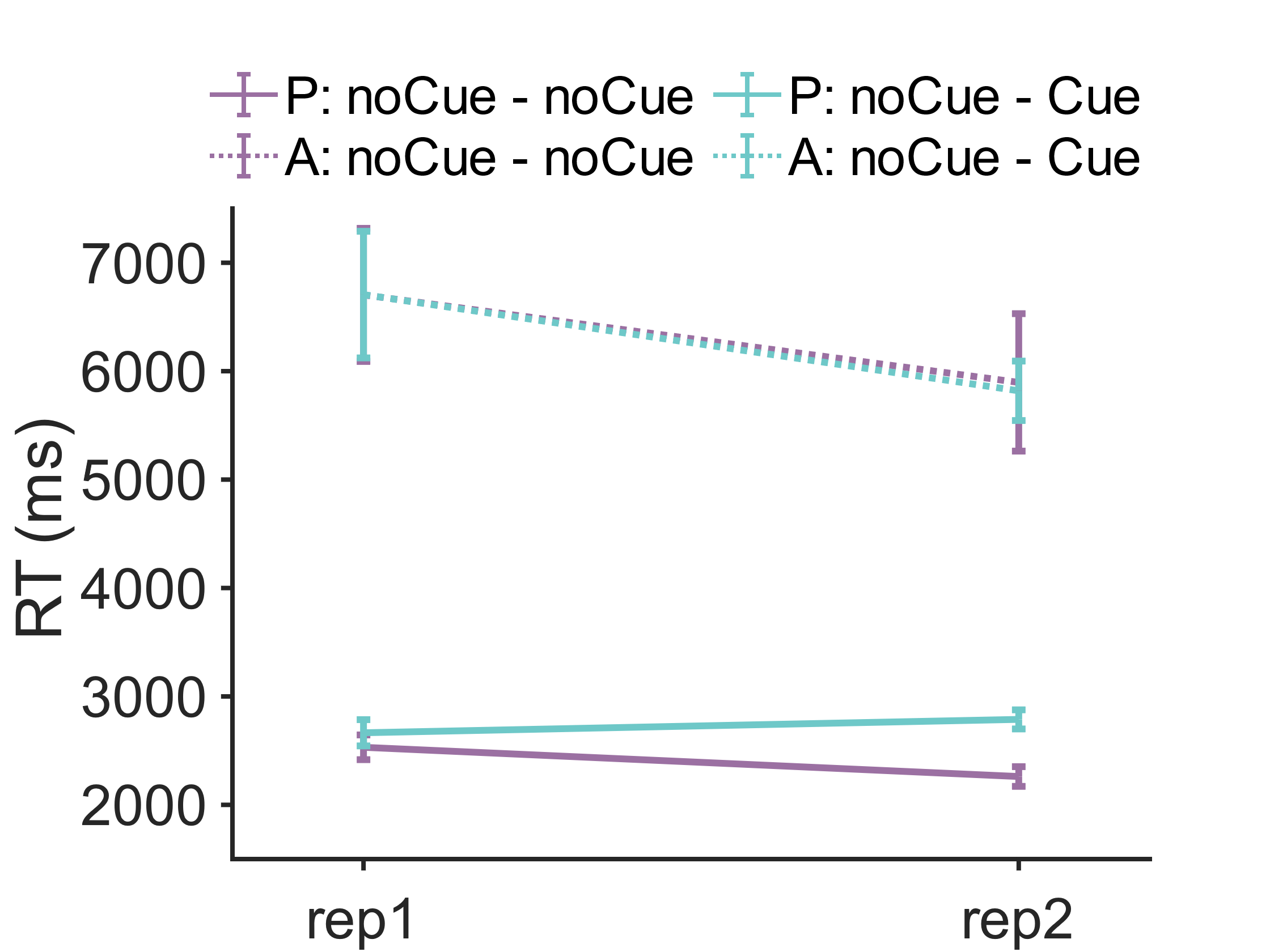


Figure S11. RTs on target present (solid line) and target absent (dashed line) trials in Experiment 2. Error bars represent $\pm1$ standard error.

Figure S12 shows the probability of the next fixation being on the target as a function of fixation-target distance in Experiment 2. As before, the difference between Figure12 and Figures 7B/7C in Wu et al.(2022) is probably due to the difference in our definitions of target fixations. If we focus on the data starting from the bin of 2 deg, Figure S12 shows the same monotonic trend as Wu et al (2022).


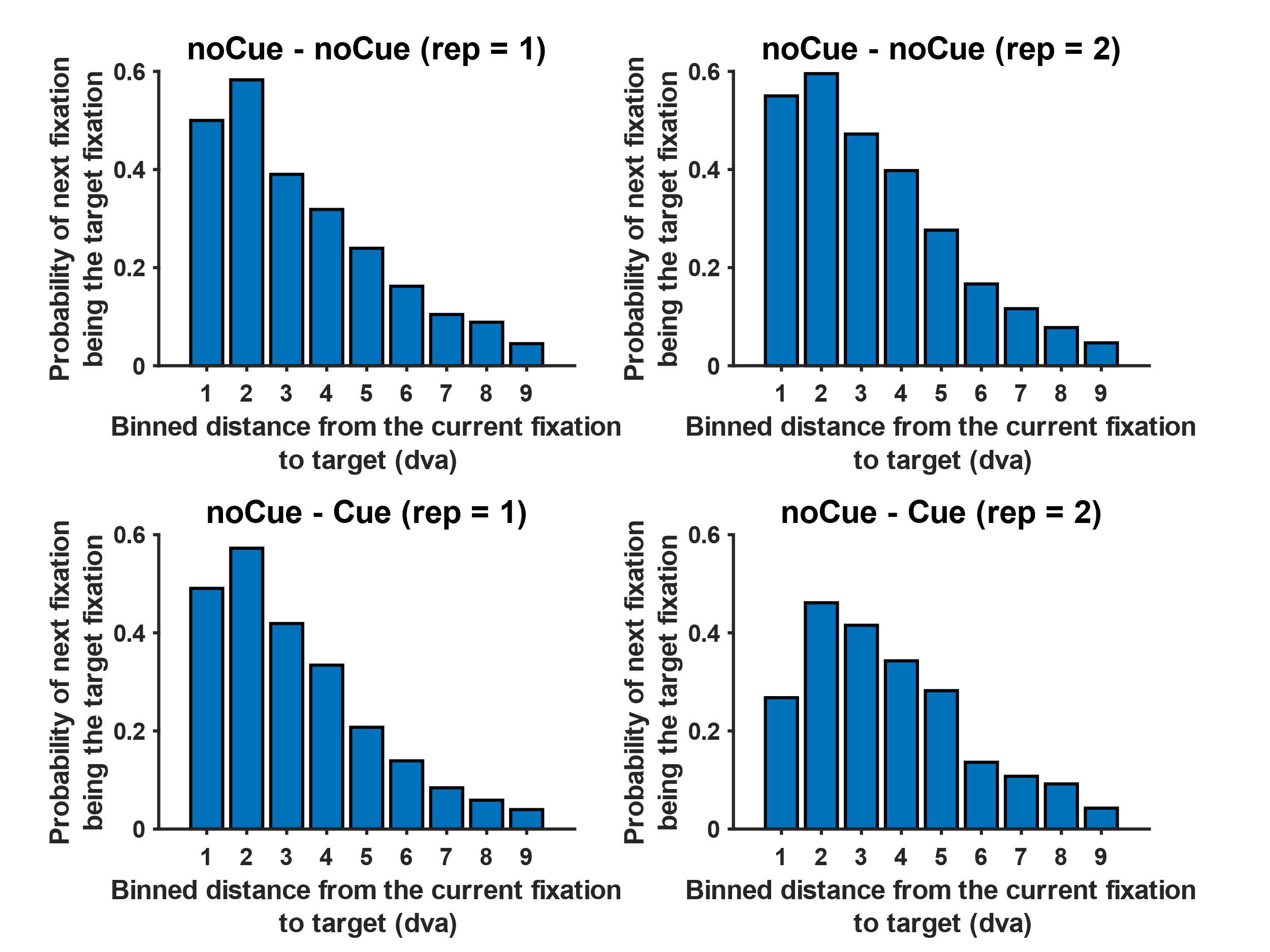


Figure S12. Probability of the next fixation being on the target as a function of fixation-target distance in Experiment 2.

Figure S13 shows the number of fixations in Experiment 2. We ran a three-way repeated measures ANOVA on the number of fixations with target presence, condition and repetition as within-subject factors. The effects of condition, repetition and target presence were all significant [condition: F(1, 17) = 5.51, p = 0.031, $\eta_{p}^{2}$ = 0.245; repetition: F(1, 17) = 6.96, p = 0.017, $\eta_{p}^{2}$ = 0.291; target presence: F(1, 17) = 69.49, p < 0.001, $\eta_{p}^{2}$ = 0.803]. The interaction between target presence and repetition was also significant [F(1, 17) = 7.32, p = 0.015, $\eta_{p}^{2}$ = 0.301]. The other two-way interactions and the three-way interaction were not significant. Then trials were split by target presence and two two-way repeated measures ANOVAs with condition and repetition as within-subject factors were conducted for target present and target absent trials separately. For target present trials, the effect of condition was significant [F(1, 17) = 44.61, p < 0.001, $\eta_{p}^{2}$ = 0.724], but the effect of repetition was not significant. The two-way interaction between condition and repetition was significant [F(1,17) = 46.83, p < 0.001, $\eta_{p}^{2}$ = 0.734], with a larger effect of condition on the second copy of stimuli. Although the presence of the Cue does not seem to increase the number of fixations on present trials, it does prevent the drop in number of fixations that can be seen for the NoCue condition. For target absent trials, the effect of condition was not significant, but the effect of repetition was [F(1, 17) = 7.40, p = 0.015, $\eta_{p}^{2}$ = 0.303]. The two-way interaction between condition and repetition was not significant. For the absent trials, the presence of a cue did not have an effect on the number of fixations.


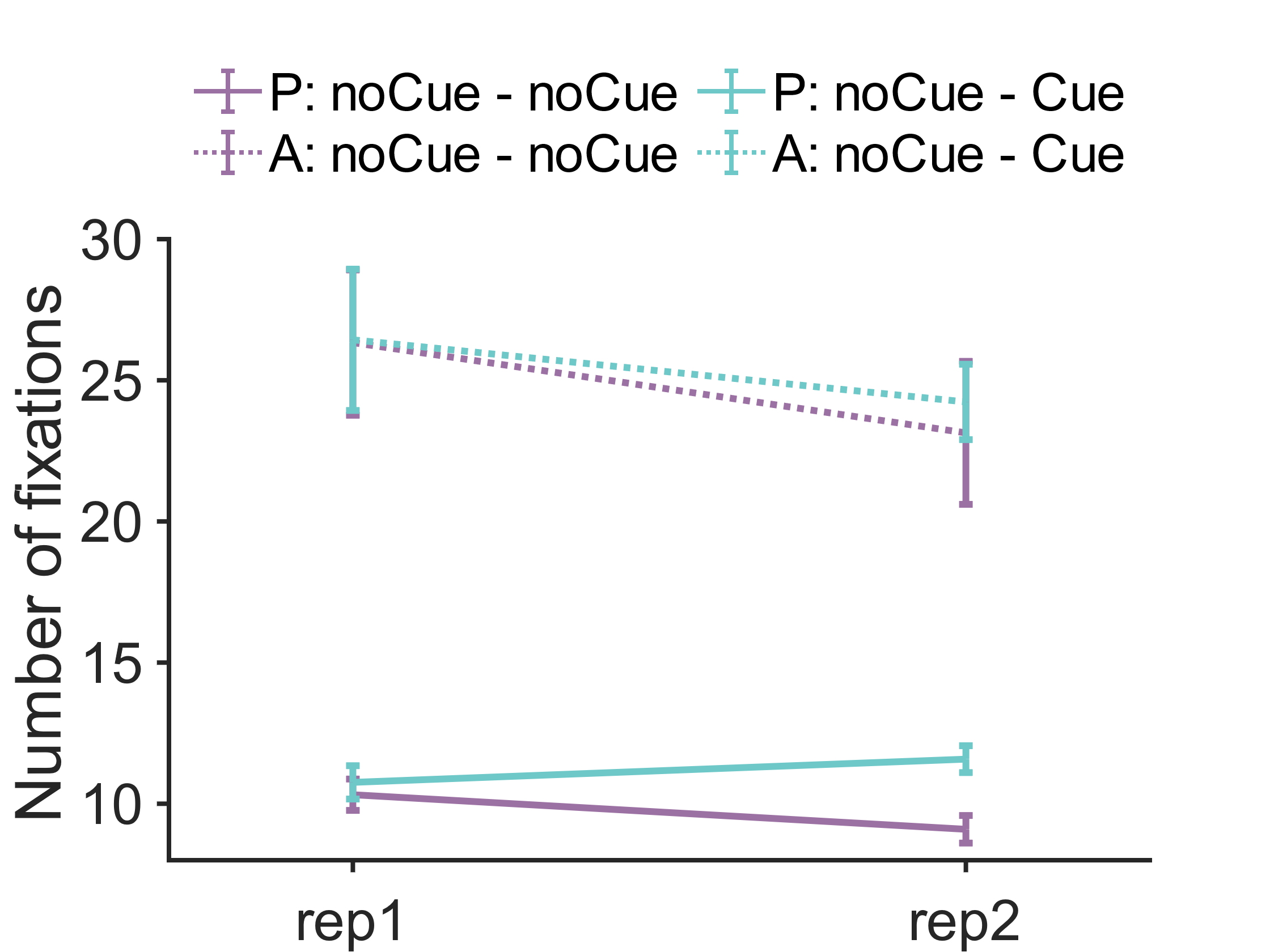


Figure S13. Number of fixations on target present (solid line) and target absent (dashed line) trials in Experiment 2. Error bars represent $\pm1$ standard error.

Figure S14 shows the fixation durations in Experiment 2. We ran a three-way repeated measures ANOVA with target presence, condition and repetition as within-subject factors on fixation durations. The effects of condition and target presence were significant [condition: F(1, 17) = 34.52, p < 0.001, $\eta_{p}^{2}$ = 0.670; target presence: F(1, 17) = 44.19, p < 0.001, $\eta_{p}^{2}$ = 0.722], but the effect of repetition was not [F(1, 17) = 2.71, p = 0.118, $\eta_{p}^{2}$ = 0.138]. The interaction between target presence and repetition was significant [F(1, 17) = 13.05, p = 0.002, $\eta_{p}^{2}$ = 0.434]. As was the interaction of repetition and condition [F(1, 17) = 41.63, p < 0.001, $\eta_{p}^{2}$ = 0.710]. The interaction between group and target presence was not significant. Neither was the three-way interaction. Because of the interaction involving target presence, we ran separate two-way repeated measures ANOVAs with condition and repetition as within-subject factors for target present and target absent trials. For target present trials, the effect of condition was significant [F(1, 17) = 139.8, p < 0.001, $\eta_{p}^{2}$ = 0.892], but the effect of repetition was not significant. The interaction between condition and repetition was significant [F(1, 17) = 43.12, p < 0.001, $\eta_{p}^{2}$ = 0.717], with a larger effect of condition on the second copy of stimuli. For target absent trials, the effects of both condition and repetition were significant [condition: F(1, 17) = 23.0, p < 0.001, $\eta_{p}^{2}$ = 0.575; repetition: F(1, 17) = 14.1, p = 0.002, $\eta_{p}^{2}$ = 0.453]. The interaction between condition and repetition was also significant [F(1, 17) = 33.5, p < 0.001, $\eta_{p}^{2}$ = 0.664], with a larger effect of condition on the second copy of stimuli. Here, it is clear that the presence of a cue reduces fixation duration, both for target present and for target absent trials.


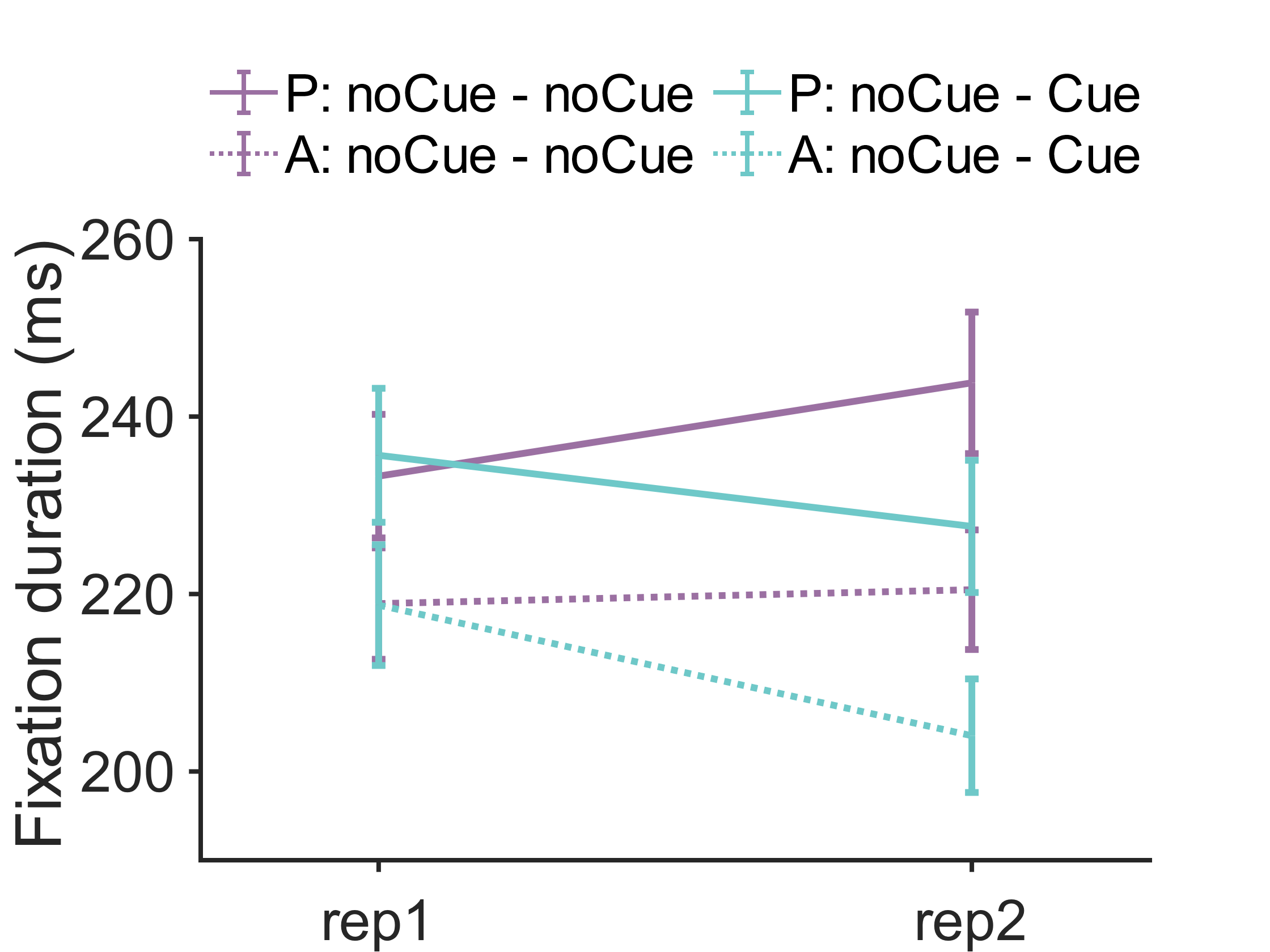


Figure S14. Fixation durations on target present (solid line) and target absent (dashed line) trials in Experiment 2. Error bars represent $\pm1$ standard error.

Figure S15 shows the average length of target saccade on target present trials in Experiment 2.


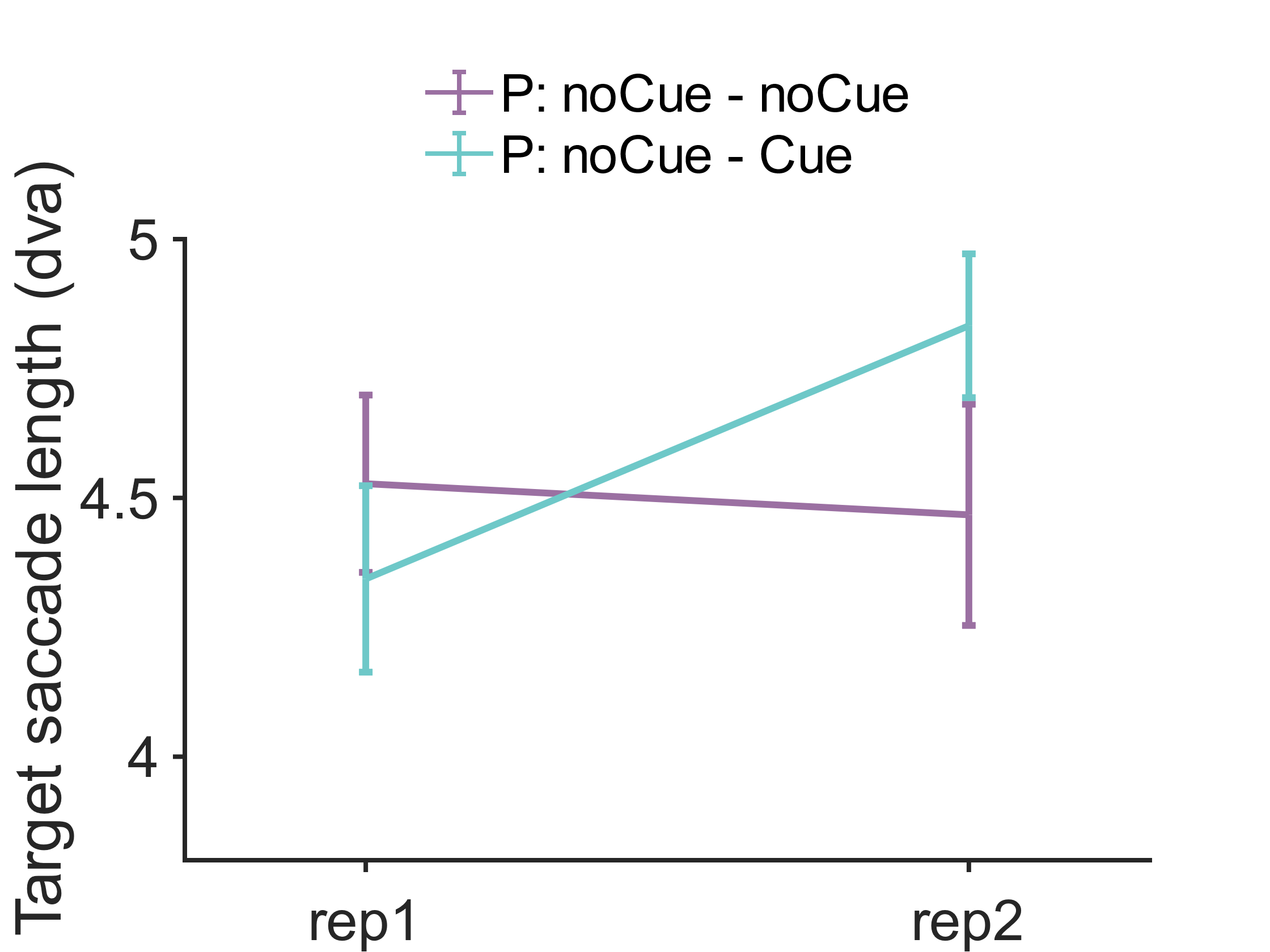


Figure S15. Target saccade length on target present trials in Experiment 2. Error bars represent $\pm1$ standard error.

Figure S16 shows the average length of search saccades in Experiment 2. We conducted a three-way repeated measures ANOVA on search saccade length with condition, target presence and repetition as within-subject factors. The effects of condition and target presence were significant [condition: F(1, 17) = 20.81, p < 0.001, $\eta_{p}^{2}$ = 0.550; target presence: F(1, 17) = 19.67, p < 0.001, $\eta_{p}^{2}$ = 0.536], but the effect of repetition was not [F(1, 17) = 2.26, p = 0.151, $\eta_{p}^{2}$ = 0.117]. The interaction between repetition and condition was significant [F(1, 17) = 21.78, p < 0.001, $\eta_{p}^{2}$ = 0.562], with a larger effect of condition on the second copy of the stimuli. All other interactions were not significant. It is clear that the cue reduces search saccade length


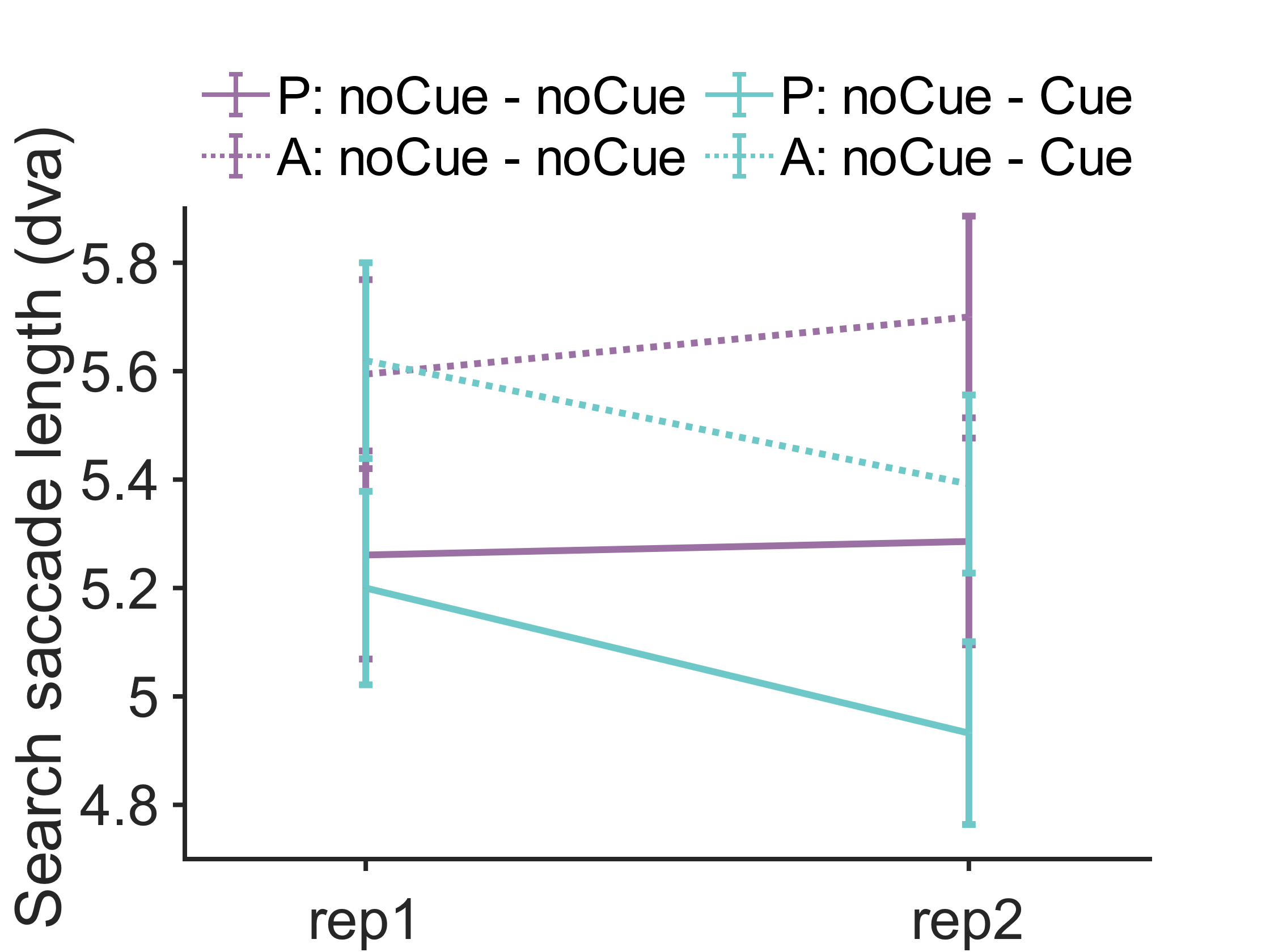


Figure S16. Search saccade length on target present (solid line) and target absent (dashed line) trials in Experiment 2. Error bars represent $\pm1$ standard error.

Figure S17 shows search saccade length on target present trials split by target contrast. For search saccade length, two participants were excluded due to empty cells. The effect of condition was significant [F(1, 15) = 6.23, p = 0.025, $\eta_{p}^{2}$ = 0.293]. The effects of repetition and target contrast were not significant. The two-way interaction between condition and repetition was significant [F(1, 15) = 6.06, p = 0.026, $\eta_{p}^{2}$ = 0.288]. The other two-way interactions and the three-way interaction were not significant. For all contrasts, the Cue reduces the length of the search saccades


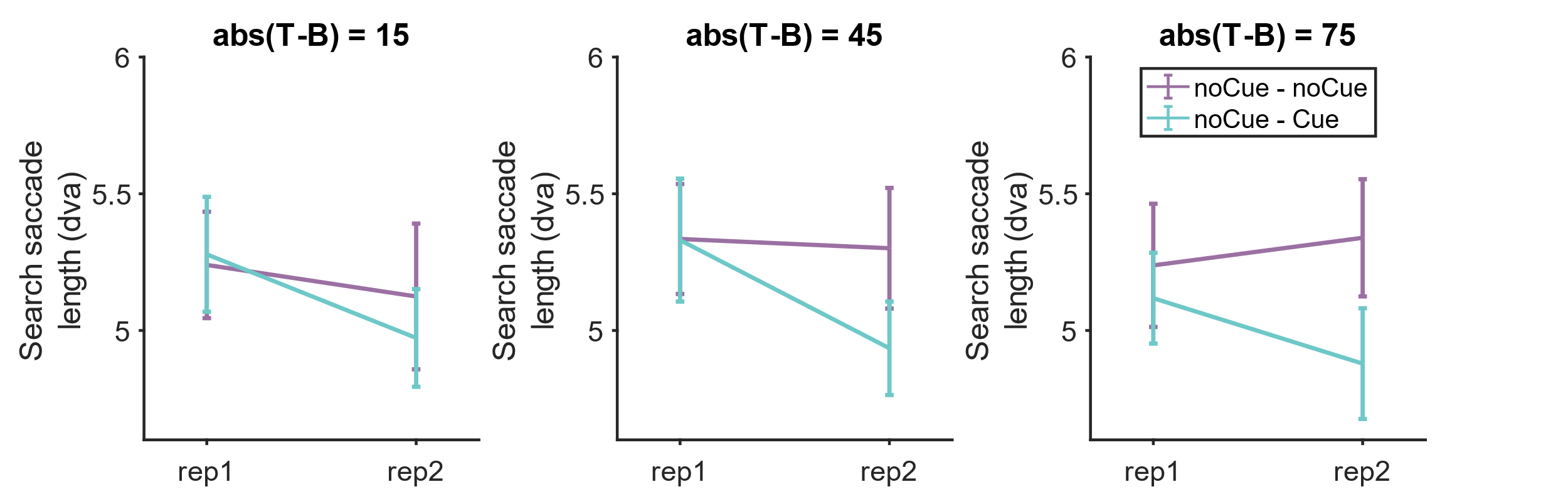


Figure S17. Search saccade length on target present trials split by target contrast in Experiment 2. Error bars represent $\pm1$ standard error.


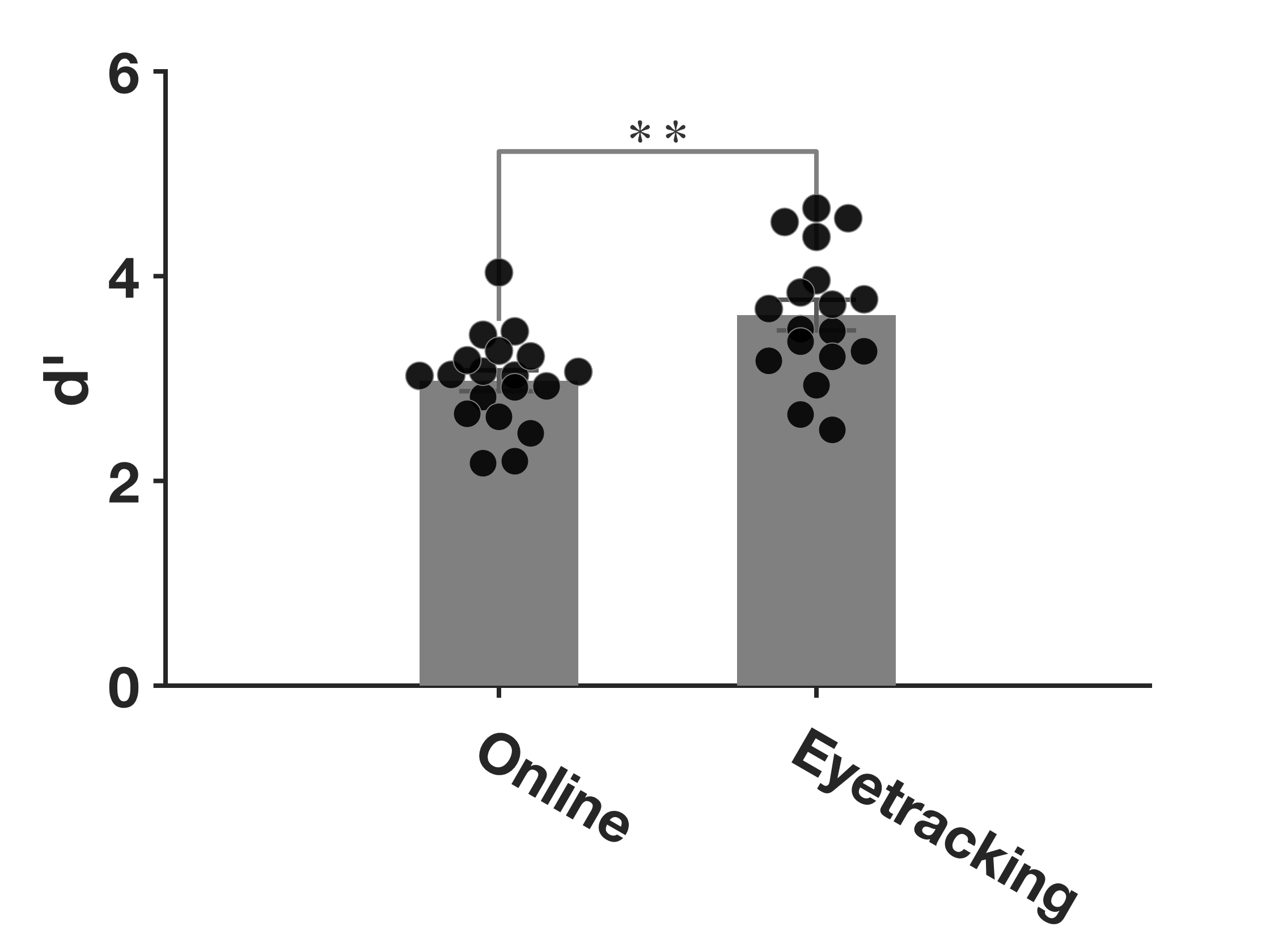


Figure S18. Comparison of d’ between the previous online Experiment 3c and the current Experiment 2.


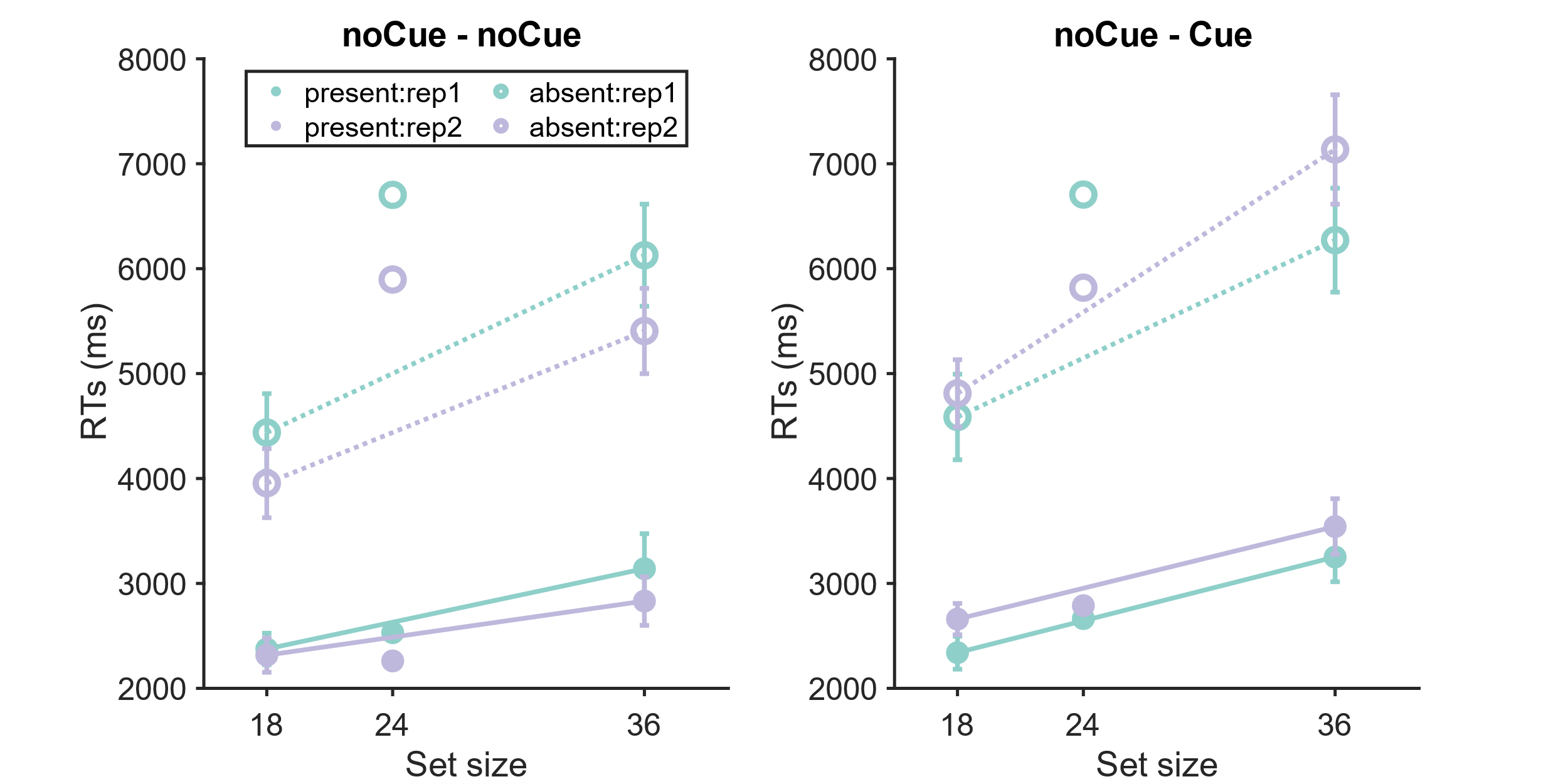


Figure S19. Comparison of RTs between the previous online Experiment 3c and the current Experiment 2. In the online experiment, the set sizes were 18 and 36. In the eye tracking experiment, the set size was 24.

**Table S1**. Results from a two-way repeated measures ANOVA on RTs for targets with contrast = 15 in online Experiment 3c.


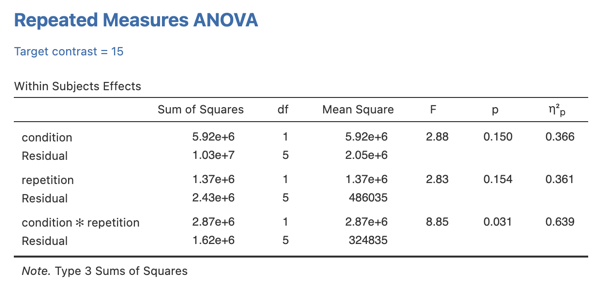


**Table S2**. Results from a two-way repeated measures ANOVA on RTs for targets with contrast = 45 in online Experiment 3c.


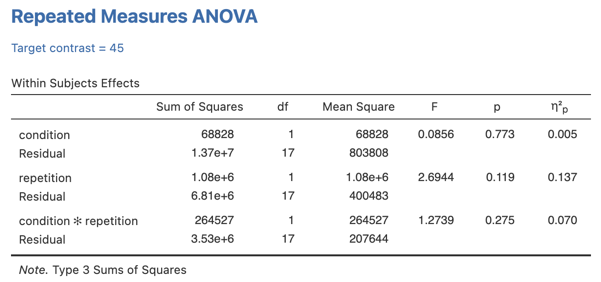


**Table S3**. Results from a two-way repeated measures ANOVA on RTs for targets with contrast = 75 in online Experiment 3c.


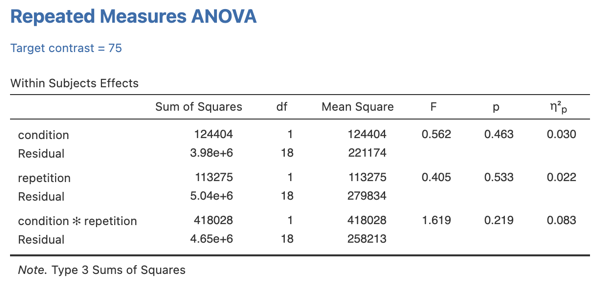


**Table S4**. Results from a two-way repeated measures ANOVA on RTs for targets with contrast = 105 in online Experiment 3c.


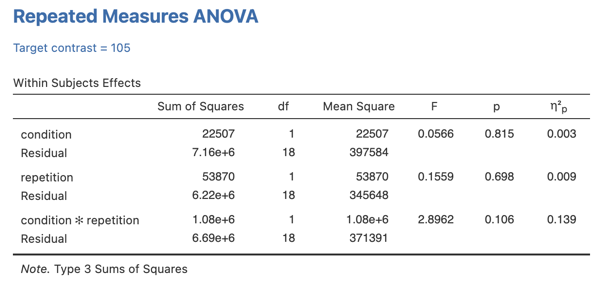

Supplement: Supplementary file 1 — Supplementary file1 (DOCX 2943 kb) [file 13414_2025_3095_MOESM1_ESM.docx]
